# Distinct water and phosphorus extraction patterns are key to maintaining the productivity of sorghum under drought and limited soil resources

**DOI:** 10.1038/s41598-025-88705-x

**Published:** 2025-02-10

**Authors:** Sara Loftus, Anna M. Sauer, Eva M. Schneider, Lalitha K. Erugoti, Murugesan Tharanya, Reimund P. Rötter, Jana Kholová, Mutez A. Ahmed, Michaela A. Dippold

**Affiliations:** 1 Biogeochemistry of Agroecosystems, University of Göttingen, Von-Thünenweg 3, 37075 Göttingen, Germany; 2 Root-Soil Interaction, TUM School of Life Sciences, Technical University of Munich, Freising, Germany; 3 Centurion University of technology and management, Paralekhemundii, Odisha India; 4 Tropical Plant Production and Agricultural Systems Modelling (TROPAGS), University of Goettingen, Goettingen, Germany; 5 Campus Centre of Biodiversity and Sustainable Land Use (CBL), University of Goettingen, Goettingen, Germany; 6 Crop Physiology Laboratory, International Crops Research Institute for Semi-Arid Tropics (ICRISAT), Patancheru, India; 7 Department of Information Technologies, University of Prague, Prague, Czech Republic; 8 Geo-Biosphere Interactions, University of Tuebingen, Tuebingen, Germany

**Keywords:** Drought, Multiple resource limitations, Phosphorus, Plant phenological development, Sorghum, Water use, Plant sciences, Biogeochemistry

## Abstract

Nutrient and water limitations contribute to yield losses in semi-arid regions. Therefore, crop rotations incorporating nitrogen-fixing legumes and drought-tolerant sorghum varieties offer a strategy to improve the utilization of scarce soil resources. Under semi-arid, field-like conditions, sorghum crop rotations with either cowpea pre-crop or fallow, including two early and three late maturing genotypes, were tested to identify stress adaptation traits of sorghum to water and phosphorus limitations. Morphological and physiological parameters were evaluated on a single-plant basis. Lower soil P content significantly delayed flowering compared to higher P levels. However, improved P availability arising from pre-crop residues reduced this effect. Mycorrhizal infection rates and root-to-shoot ratios were positively correlated with panicle N and P content at anthesis under low P conditions. Although drought significantly impacted yield, early maturing genotypes with the highest reduction in shoot biomass and reduced water use before flowering, could sustain yield production. Early-maturing genotypes characterized by high root-to-shoot ratios, rapid AMF establishment, and reduced water use before flowering exhibit a strong potential for maintaining yield and biomass production on nutrient-poor soils in semi-arid regions. Such genotypes conserve water before flowering and thus can alleviate post-flowering water stress, ensuring adequate P uptake despite low soil P availability.

**Supplementary Information:**

The online version contains supplementary material available at 10.1038/s41598-025-88705-x.

## Introduction

Water scarcity^1^ and low soil fertility^2^ compromise crop production in semi-arid (sub-) tropical regions. According to future climate projections, many semi-arid regions will become even drier with climate change^3^ and, combined with ongoing land degradation, will further reduce the area suitable for crop production^4^. At the same time, the global population continues to increase, with the highest growth projected for sub-Saharan Africa, where the population is estimated to exceed 2 billion by 2050^5^. Sustainable agricultural practices are essential to maintaining food security and environmental resilience. The implementation of legume crop rotations in combination with drought-adapted staple crops such as sorghum can harness numerous benefits, including improved soil health, crop productivity, and reduced environmental impacts^6^.

Many subsistence farmers in the semi-arid regions in Africa and Asia grow sorghum (*Sorghum bicolor* [L.] Moench) for human consumption^7^. Sorghum is the fifth most important staple crop in the world^8^. Apart from human nutrition, sorghum serves as livestock fodder^9^, bioenergy production^10^, and the production of traditional beverages in some regions of Africa^11^. Sorghum is an annual short-day member of the family *Poaceae*, which originates from Africa and is predominantly self-pollinating. With a C4 photosynthetic pathway and a thick wax leaf layer sorghum is adapted to semi-arid conditions^12^. In India, sorghum cultivation in the post-rainy season is common practice, utilizing stored soil moisture to meet the plant’s water demand for the growing season^13^. Although sorghum tolerates drought conditions better than most other crops, water stress remains the most significant factor limiting crop yields under these cultivation systems^14^. Drought can occur at all crop development stages and, therefore, causes many complex plant responses that cannot be jointly targeted within one breeding program^15^. The categories of the drought adaptation mechanisms and the related traits are drought avoidance, escape, and tolerance, each requiring specific combinations of diverse morphological and physiological adaptations^16^. Mainly, water conservation and efficiency of water use are related to how biomass production is adapted to soil moisture availability under water-scarce conditions^15^. The degree of conserving soil water can be estimated from transpiration rates normalized by leaf area^17^. Early flowering^18^, fast maturity, and plasticity of leaf area development are characteristics of drought-adapted sorghum varieties^19^. The high genetic diversity of sorghum for early and mid-season drought stress adaptations^20^ facilitates the development of resilient and adaptable varieties to meet the challenges of drought-prone environments.

Mineral fertilizers are used to a very limited extent in smallholder crop production systems. Cash crops are mainly fertilized during the rainy season, while sorghum is primarily grown in the post or the short rainy season with low or no inputs in India^21^ and sub-Saharan Africa^22^. Prevailing, soil types like Alfisols have little organic matter and plant-available nutrient contents^23; 24^, especially plant-available phosphorus (P)^25^.

Furthermore, crop rotations with legumes are a common practice, and due to their ability to fix atmospheric nitrogen, the use of nitrogen fertilizers could be partly reduced. Across agroecological zones of sub-Saharan Africa legume crop rotations significantly increased cereal yields of maize, sorghum and millet, although the response of sorghum and millet was less pronounced compared to maize^6^. In particular, subsistence farmers rely on low cost management practices. In that context, legume crop rotations affect the productivity of crop rotations and farmers’ revenues, as expensive nitrogen fertilizers can be partly substituted. Decomposing root residues and the respective root channels serve as nutrient, organic carbon, and microbial activity hotspots^26^. Further implications for soil health and nutrient cycling after legume cropping could be observed. Lower infection rates with the parasitic plant Striga^6^, enhanced mycorrhizal infections, and increased phosphatase activity resulted in higher N and P contents of the subsequently cultivated sorghum^27^.

Since water shortages and low plant-available P decrease sorghum yields^28^, evaluating of the performance of different sorghum varieties under multiple resource limitations is essential to identify varietal traits that may be useful in plant breeding programs.

To acquire knowledge about specific sorghum traits that mitigate stress under multiple resource (i.e. water, P and N) limitations, a four-factorial experiment was conducted. Two water levels (well-watered –WW and water-stressed –WS), two P-levels (high P and low P), two different crop rotations (legume pre-crop and fallow) and five sorghum genotypes (two early maturing and three late maturing genotypes) were tested.

The study aims were (i) to examine the performance of early and late maturing sorghum genotypes, (ii) to measure if different soil P availabilities influence water-stress mitigation, and (iii) to determine whether a crop rotation with legume pre-crops has a positive influence on sorghum’s nutrient status and water uptake by enhancing the nutrient uptake from organic residues under limited water and nutrient availability. The resulting hypotheses were that (i) early maturing genotypes perform better under water limiting conditions, (ii) that a higher P availability leads to yield increase under drought conditions, (iii) that genotypes that use the residual nutrients from a legume crop have an improved nutrient supply which leads to higher biomass and yield production, especially under concomitant P and water limitations.

## Results

### Sorghum biomass and grain yield

**Fig. 1:**
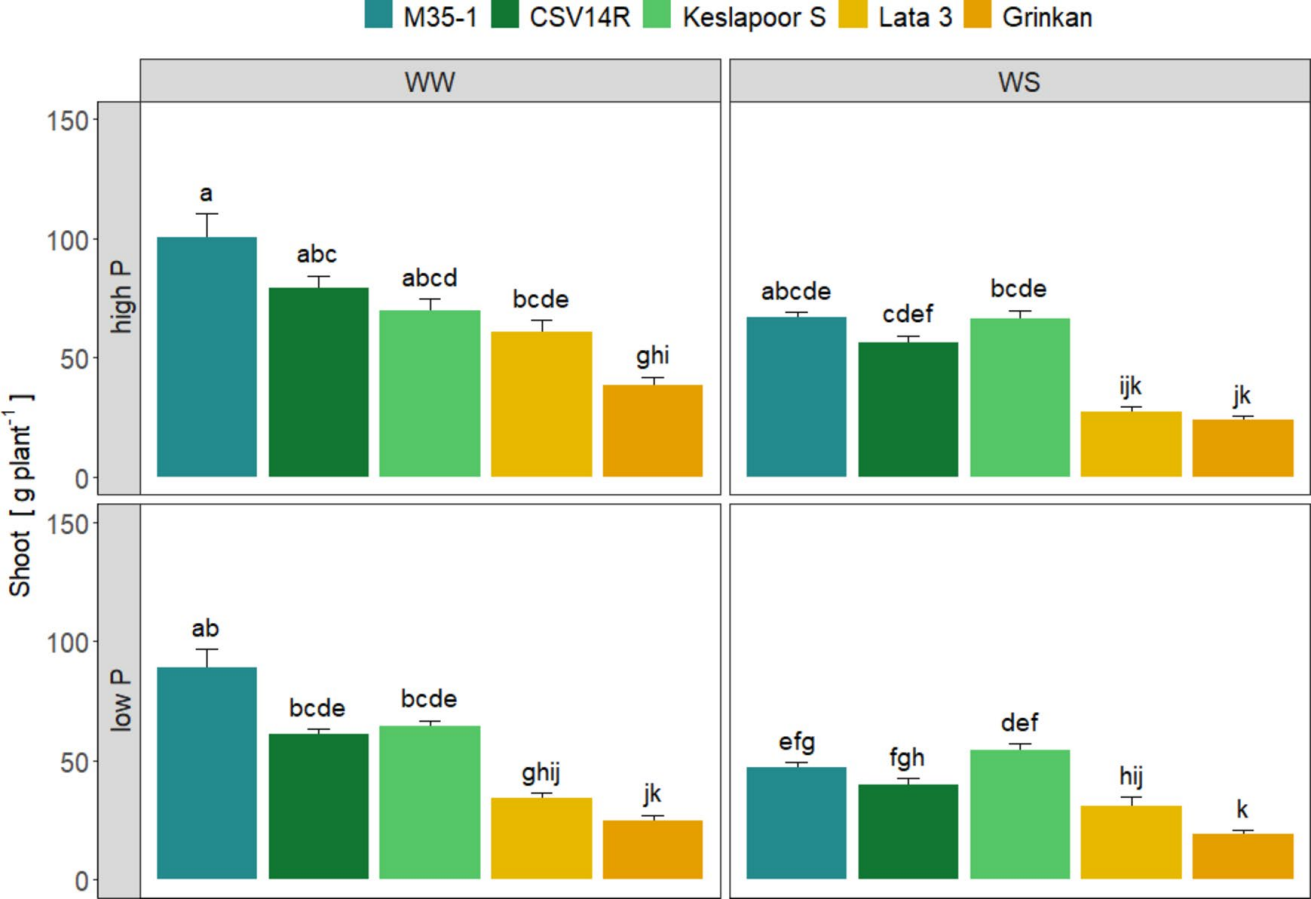
Shoot biomass dry weight of five sorghum genotypes grown under two water levels well-watered (WW) and water-stressed (WS) conditions and two P levels, high and low P. Late maturing genotypes are represented by CSV14R, Keslapoor S and M35-1 and early maturing genotypes are represented by Lata 3 and Grinkan. Data show arithmetic means (*n* = 8–10) and standard error (SE) of the mean. The different letters indicate significant differences (*p* < 0.05, Tukey post-hoc test) of log-transformed data.

Figure 1 shows the effects of different P levels and water conditions on the shoot biomass of five sorghum genotypes (M35-1, CSV14R, Keslapoor, Lata, and Grinkan). Although all factors affected shoot biomass (genotype (p = < 2.2e−16), P level (p = < 6.4e−12), water availability (p = < 2.2e−16), crop rotation (p = < 1.1e−06)) only the significant factor combination genotype: P level: water level (*p* = 0.0013) is shown. The four factorial treatment interactions were insignificant, as shown in the supplement (Supplement, Fig. S1, Table S1).

The late maturing genotype M35-1 showed the highest shoot biomass with a range of 47.1–100.4 g plant^− 1^. The genotype CSV14R showed a similar shoot biomass response to water and nutrient availability as M35-1 but with a lower range between 39.7 and 79.0 g plant^− 1^. The biomass production of Keslapoor S was lower (54.3–69.6 g plant^− 1^) compared to M35-1 but was least affected by water and P limitations. The late maturing genotypes generally showed a higher shoot biomass production than the early maturing genotypes. The early maturing genotype Grinkan had the lowest shoot biomass production (19.0–38.4 g plant^− 1^), while the second early maturing genotype Lata 3 (27.0–60.9 g plant^− 1^) had either a similar shoot production as Grinkan or an intermediate shoot production between Grinkan and the other late maturing genotypes.

**Fig. 2:**
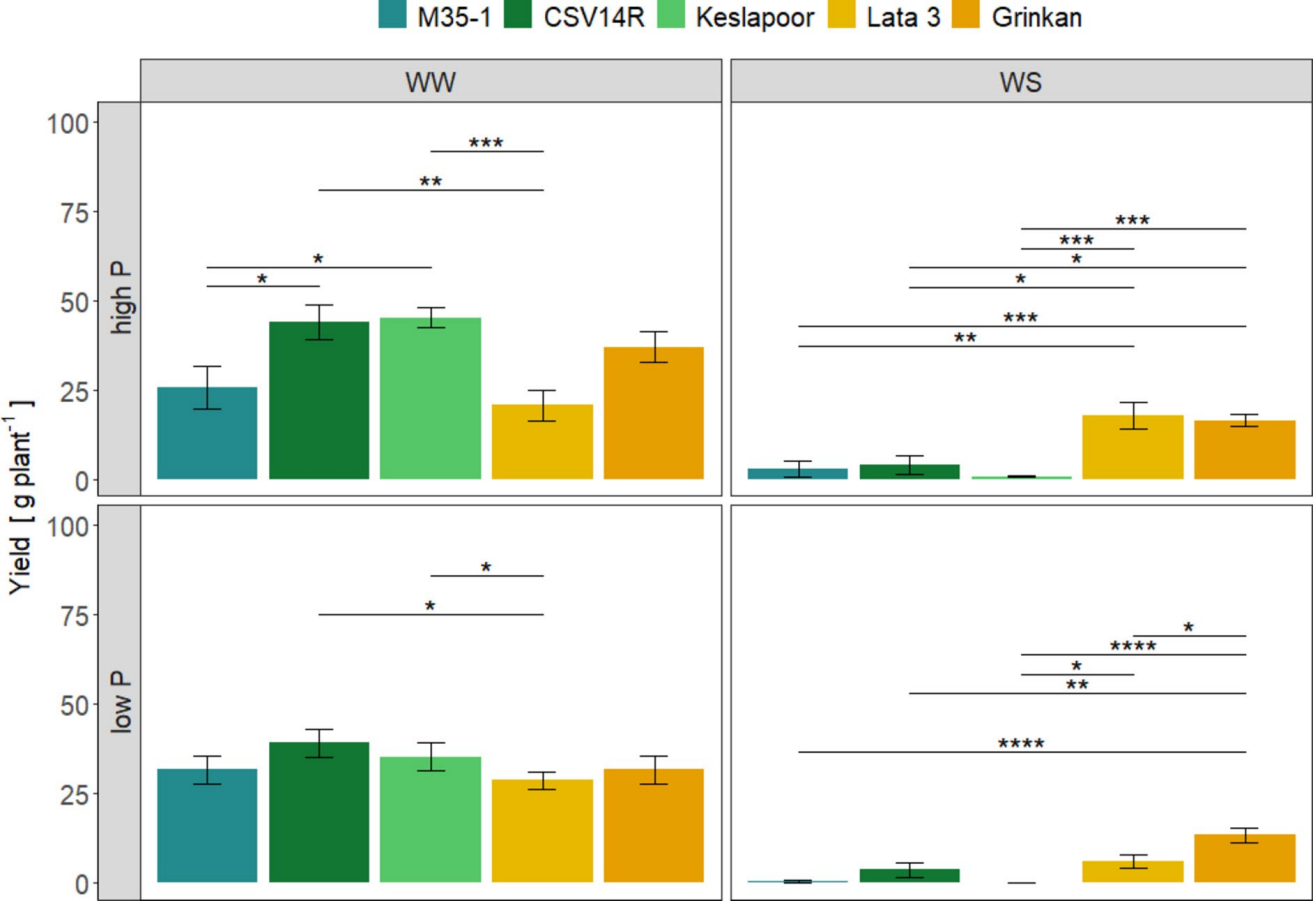
Yield of five sorghum genotypes grown under two water levels, well-watered (WW) and water-stressed (WS) conditions and two P levels, high and low P. Late maturing genotypes are represented by CSV14R, Keslapoor S and M35-1 and early maturing genotypes are represented by Lata 3 and Grinkan. Data show arithmetic means (*n* = 8–10) and standard error (SE) of the mean. Asterisks show significant differences between the genotypes (*p* = 0.05, Dunn’s test) with the factor genotypes as pairwise comparisons.

The water availability mainly influenced sorghum yield production, and drought had an overall negative effect (Fig. 2). In fact, only Lata 3 could maintain yield levels (17.8–20.6 g plant^− 1^) under high P irrespective of water levels (Supplement, Table 1). Comparing the yield production between the genotypes, Keslapoor S (45.0 g plant^− 1^) achieved the highest yields, followed by CSV14R (44.0 g plant^− 1^) and Grinkan (37.0 g plant^− 1^) under high P availability and sufficient water supply (Fig. 2). A lower soil P availability resulted in an approximation of yield production between the genotypes under sufficient water supply (Fig. 2). Furthermore, P availability did not affect yield production under sufficient water supply, while under WS only Keslapoor S and Lata 3 displayed a significant yield decrease from high to low P levels (Supplement, Table S3).

Under WS conditions, Grinkan was the most productive genotype with 13.4–16.5 g plant^− 1^, followed by Lata 3 with 6.03–17.8 g plant^− 1^. Keslapoor S and M35-1 produced almost no yield under drought conditions (Fig. 2).

### Effect of water use till flowering on HI

**Fig. 3:**
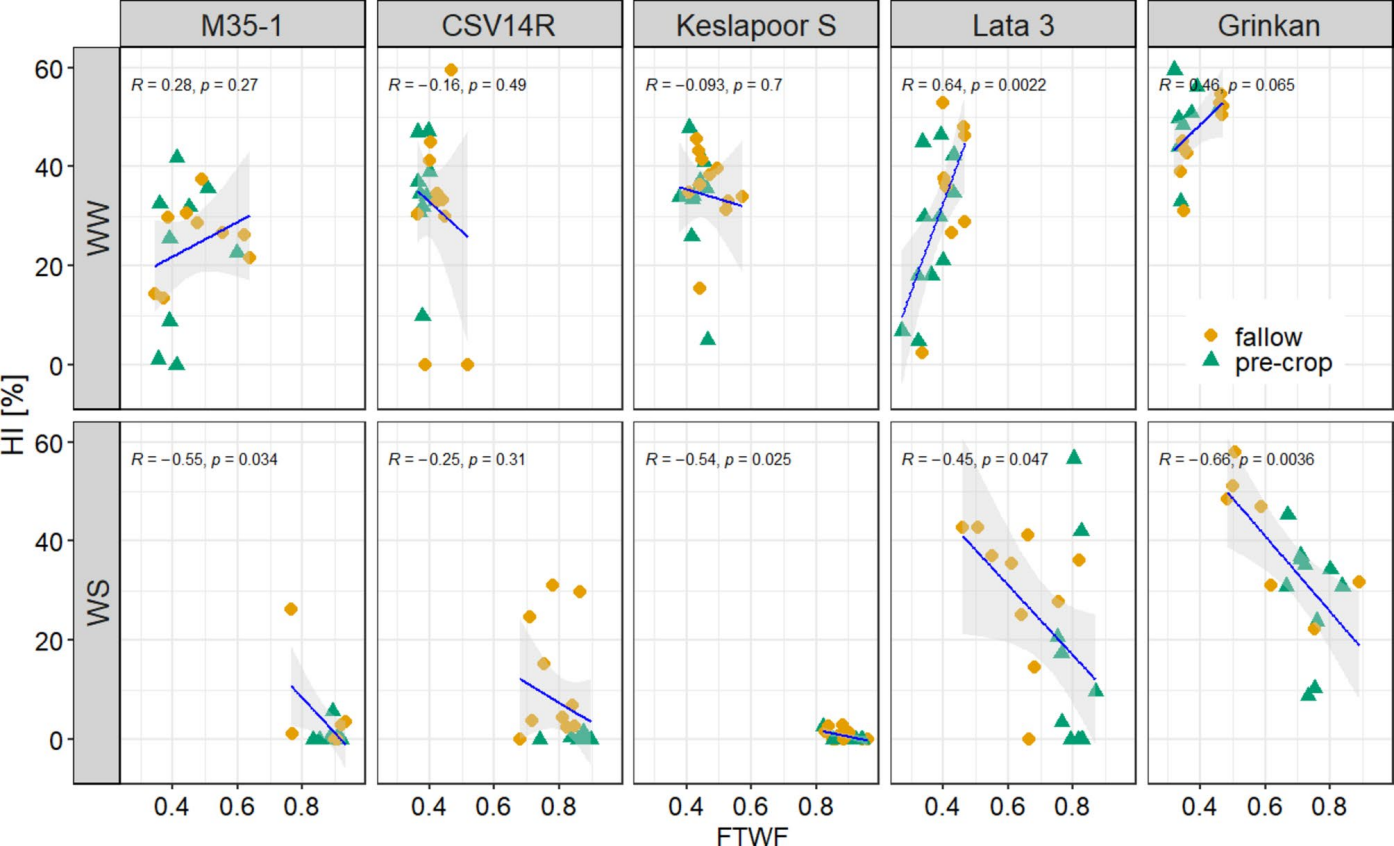
Relation between the Harvest Index (HI in %) and the Fraction of Transpired Water till Flowering (FTWF) separated between the genotypes (late maturing genotypes are represented by CSV14R, Keslapoor S and M35-1 and early maturing genotypes are represented by Lata 3 and Grinkan) and water levels, well-watered (WW) and water-stressed (WS) conditions. Orange circles represent the mineral and the green triangles the organic N sources. The Spearman method was used to calculate the correlation coefficient R and p-values.

The relation between Fraction of Transpired Water till Flowering (FTWF) and Harvest Index (HI in %) was affected by water availability and sorghum genotypes (Fig. 3). The early maturing genotypes showed a positive correlation under WW (Lata 3, *R* = 0.64, *p* = 0.0022, Grinkan, *R* = 0.46, *p* = 0,065), while under WS the relation was negative (Lata 3, *R*=−0,45, *p* = 0.047, Grinkan, *R*=−0.66, *p* = 0,0036). Especially early maturing genotypes grown in tubes with organic residues and late maturing genotypes under WS had FTWF values higher than 0.7 and showed a negative relation with HI. Compared to the mineral N fertilization, Lata 3 and Grinkan had a significant FTWF increase of 16% and 19% in the organic N treatment under WS (Supplement Fig. S3). Under WS, the early maturing genotypes Grinkan and Lata 3 had FTWF values between 0.53 and 0.76 and 0.64–0.82, respectively. Meanwhile the FTWF of the late maturing genotypes CSV14R, Keslapoor S, and M35-1 ranged between 0.76 and 0.86, 0.87–0.90 and 0.86–0.89 under WS, respectively (Supplement, Fig. S3).

### Transpiration normalized by leaf area Tn

**Fig. 4:**
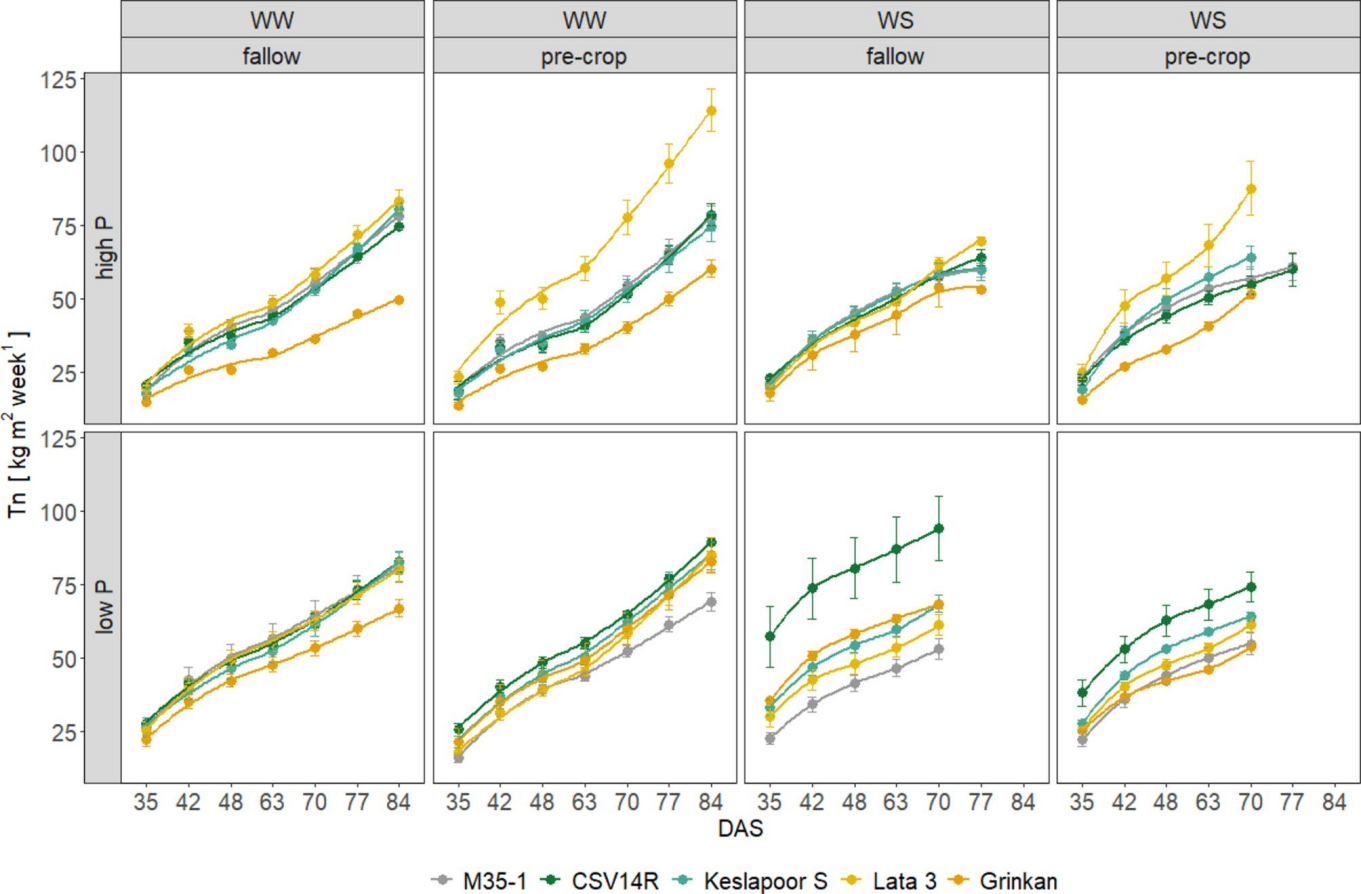
Normalized transpiration by leaf area (Tn) (kg m^-2^ week^-1^) over time (DAS) of five sorghum genotypes grown under two water levels, well-watered (WW) and water-stressed (WS) conditions, two P levels, high and low P and supplied with either the fallow or the pre-crop crop rotation. Late maturing genotypes are represented by CSV14R, Keslapoor S and M35-1 and early maturing genotypes are represented by Lata 3 and Grinkan. Data show arithmetic means (*n* = 3–5) and standard error (SE) of the mean.

Grinkan had a trend towards lowest T_n_ under high P at both water levels compared to the other genotypes, while this trend diminishes under low P (Fig. 4). Lata 3 had the trend of having highest T_n_ values under high P levels in those cylinders with cowpea pre-crop cultivation compared to the other genotypes. The T_n_ of the other genotypes ranges within the levels of Lata 3 and Grinkan, except for CSV14R in the low P, WS, fallow treatment with very high T_n_ values.

### Phenological development and resource acquisition

**Fig. 5:**
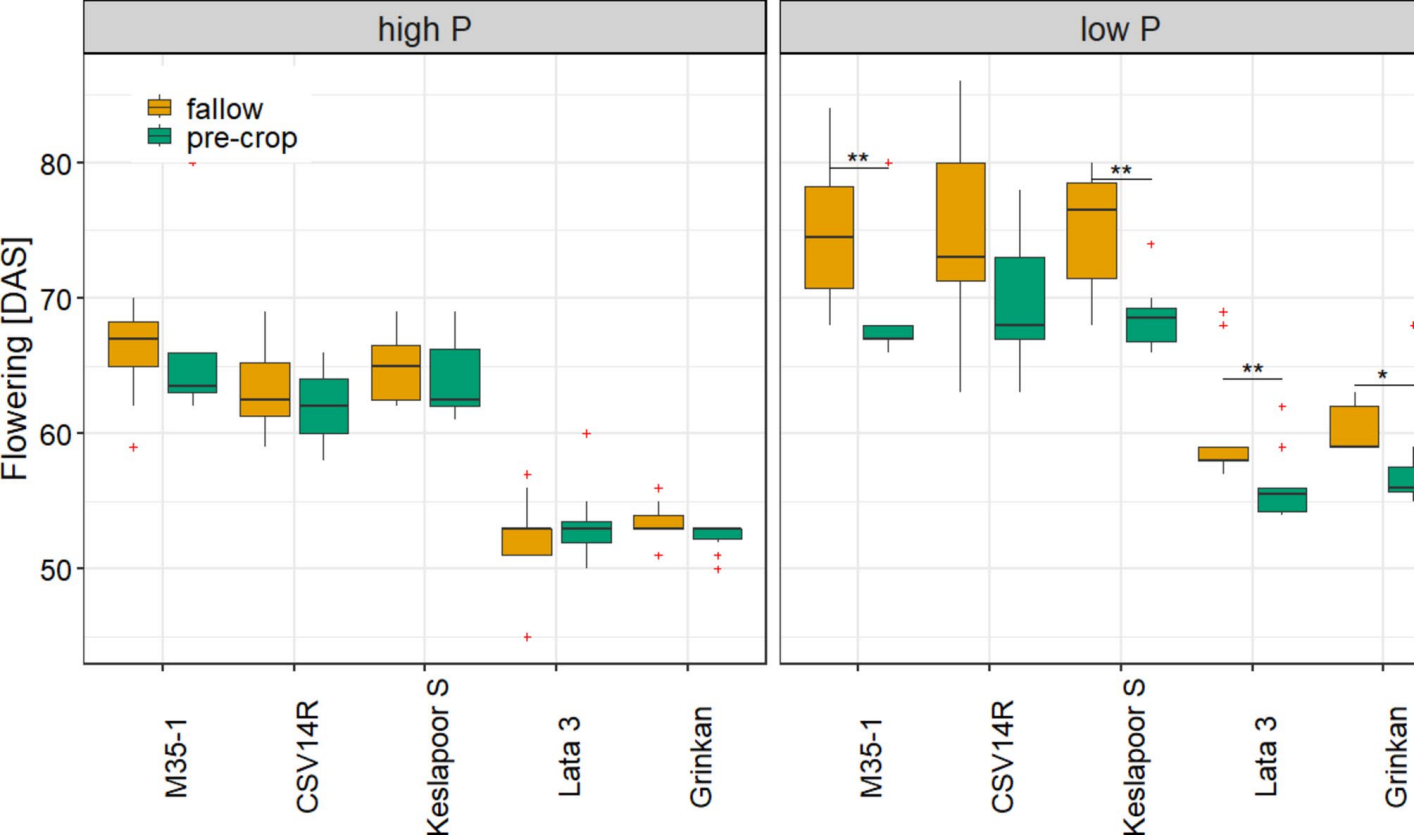
Duration of flowering time (in DAS = days after sowing) of 5 sorghum genotypes grown under (**a**) high and (**b**) low P soil contents. The data is averaged over water levels (*n* = 10). Asterisks show significant differences between the crop rotations (*p* = 0.05, Dunn’s test).

On high P soil the early maturing genotypes flowered on average at 52.7 days after sowing (DAS), which was on average 11.7 days earlier, than the late maturing genotypes (DAS 64.3). On low P soil, the difference was on average 13.4 days (DAS 58.6 and DAS 72). Low soil P contents significantly delayed plant development compared to high P levels, as flowering was delayed by 5.8 days for early and 7.8 days for late maturing genotypes. However, the pre-cropping per se did not affect the flowering time point of plants grown on soils with high P levels. The presence of belowground organic residues significantly accelerated the development of all genotypes grown under low P, detectable by a 3–6 days earlier flowering time point (Fig. 5). Drought had a negative impact, specifically for the late maturing genotypes, as approx. 5% of the plant individuals could not produce any flower irrespective of the soil P level, while the early maturing genotypes could maintain flower development.

At stem elongation, the plants grown under low P levels had on average approx. 39% lower shoot P content than under high P levels, irrespective of the genotype (Table 2). Apart from that, the root-to-shoot ratios were not significantly different between the P levels. However, the early maturing genotypes showed a higher root-to-shoot ratio trend as well as higher mycorrhizal infection rates (Table 1). Furthermore, high and positive correlation coefficients at stem elongation are shown in the correlation plots in the Supplement. Fig. S7A, B illustrate the positive interaction of above and belowground sorghum traits such as shoot biomass, root biomass, root length (Rlength), root surface area (SurfArea), as well as with transpiration. Furthermore, in the early stage, roots and root growth parameters show a negative trend with mycorrhization (Supplement, Fig. S7A, B).

The early maturing genotypes had a significantly higher root-to-shoot ratio (approximately 40%) at anthesis than the late maturing ones (Table 2). Also, the mycorrhizal infection rate of the early maturing genotypes increased significantly under low P compared to high P levels (Table 1).

Negative correlation coefficients at anthesis indicate the start of resource partitioning in different plant parts shown, e.g. by the P partitioning between vegetative (shoot P, root P) and generative (flower P) plant organs (Supplement, Fig. S8). While shoot biomass was still positively correlated with root parameters and transpiration, an increasing root-to-shoot ratio had a highly significant and negative correlation with transpiration (*r* =-0.7, *p* = 0.0009). Under low P, mycorrhizal infection rates were positively correlated with shoot P content at anthesis for early (*r* = 0.99, *p* = 0.01) and late maturing genotypes (*r* = 0.96, *p* = 0.0019). At anthesis, the mycorrhization infection rates of early maturing genotypes were significantly higher at low P compared to high P (*p* = 0.0433). Furthermore, mycorrhization showed a positive and significant correlation with panicle P and N content under low P (Supplement, Fig. S8B). However, mycorrhiza did not display an impact on panicle P or N contents for sorghum grown on the high P soil but for shoot P (Supplement, Fig. S8A). The root-to-shoot ratio showed a positive correlation with P and N panicle content, as well as panicle weight (Supplement, Fig. S8B). Plants grown on high P soil showed a negative correlation of root and shoot-related parameters with panicle biomass and P content (Supplement, Fig. S8A).

Under low P conditions, the root C/N ratio waspositively correlated with shoot biomass and transpiration (Supplement, Fig. S7B). Early and late maturing genotypes exhibited contrasting biomass partitioning patterns among the plant organs. All genotypes showed an increasing root-to-shoot ratio during plant development until flowering. However, this ratio declined after flowering for the early maturing genotypes, while it remained constant for the late maturing genotypes (Table 1). Throughout the sorghum development, the shoot P content significantly decreased from stem elongation to maturity under both P and water levels (Table 1). At maturity, however, shoot P content was higher under WS compared to WW conditions (Table 2).

**Table 1:** Results of sorghum for root-to-shoot ratio, root biomass, shoot biomass and total N and P contents (TN and TP) of roots and shoots and arbuscular mycorrhiza fungi (AMF) infection rates after the additional destructive harvest time points (stem elongation, anthesis and maturity).

	Development	High P							Low P						
	Stage	Early			Late			p.sig.	early			late			p.sig.
		Mean		SE	Mean		SE		Mean		SE	Mean		SE	
Root-to-shoot ratio	Stem Elongation	0.140	a	0.050	0.156	a	0.028		0.255	a	0.014	0.147	a	0.037	
	Anthesis	0.433	ab	0.010	0.286	ab	0.048	*	0.455	ab	0.058	0.245	a	0.021	*
	Maturity	0.233	a	0.029	0.244	a	0.020		0.319	a	0.013	0.347	ab	0.056	
Root [g]	Stem Elongation	2.34	a	1.08	2.44	a	0.77		4.3	a	0.39	2.60	a	0.79	
	Anthesis	8.14	b	1.38	7.55	ab	1.54		6.1	ab	0.96	9.53	b	0.73	*
	Maturity	8.72	b	2.01	11.20	b	1.64		7.11	b	0.62	16.50	b	4.66	*
Shoot [g]	Stem Elongation	16.3	a	2.77	13.5	a	2,52		16.8	ab	0.83	15.4	a	3.65	
	Anthesis	18.9	a	3.33	27.3	ab	4,07		13.6	a	0.63	40.7	b	5.17	*
	Maturity	37.0	b	7.53	45.1	b	3,22		22.7	b	3.61	44.6	b	5.56	*
TP root [mg plant¯¹]	Stem Elongation	4.69	ns	2.76	2.47	a	0.73		4.22	ab	0.61	2.99	a	0.99	
	Anthesis	6.30	ns	0.86	5.51	b	1.18		5.12	b	0.81	4.54	ab	0.65	
	Maturity	3.49	ns	1.48	6.21	b	0.47		2.76	a	0.50	6.58	b	1.62	*
TP shoot [mg plant¯¹]	Stem Elongation	46.9	a	3.35	42.6	a	6.07		31.2	ns	1.21	30.5	ns	7.57	
	Anthesis	52.8	ab	3.41	63.4	b	4.56		31.6	ns	4.49	47.7	ns	3.88	*
	Maturity	64.2	a	7.51	64.6	b	7.02		24.8	ns	6.96	40.8	ns	6.40	
TN root [mg plant¯¹]	Stem Elongation	43.8	ns	20.0	33.5	a	10.3		62.3	ns	7.48	42.7	ns	13.1	
	Anthesis	67.3	ns	11.0	51.6	a	12.5		44.8	ns	3.88	72.9	ns	7.62	
	Maturity	81.6	ns	23.5	104	b	8.47		35.8	ns	21.2	57.4	ns	19.1	
TN shoot [mg plant¯¹]	Stem Elongation	498.0	ns	187.0	409	ns	69.5		415.0	b	10.9	393	ab	87.7	
	Anthesis	300.0	ns	40.8	402	ns	51.1		273.0	ab	35.8	470	b	38.4	
	Maturity	289.0	ns	67.1	356	ns	79.3		207.0	a	75.3	328	a	50.2	
AMF infection rates [%]	Stem Elongation	3.50	ns	1.67	2.39	a	0.61		6.14	ab	2.86	5.98	ns	2.76	
	Anthesis	5.01	ns	2.36	13.1	b	3.76		13.5	b	2.48	9.77	ns	4.06	
	Maturity	9.26	ns	8.14	12.0	ab	3.23		3.34	a	1.12	17.3	ns	7.88	
		n=4			n=4				n=6			n=6			

Significance letters show significant differences (Dunn’s test, *p* < 0.05) between growth stages for early or late-maturing genotypes separately for each P level. In addition, the asterisks indicate significant differences between the early and late maturing genotypes separately for each growth stage and P level (0.05, 0.01, 0.001; *, **, ***).

**Table 2:** Mean shoot P contents after additional destructive harvest time points (stem elongation, anthesis and maturity).

Development	Shoot *P* (mg g-1)									
	WW					WS				
Stage	High P		Low P			High P		Low P		
Stem elongation	3.29	b	2.01	b	***	–		–		
Anthesis	2.8	b	1.71	ab		2.76	a	1.6	a	*
Maturity	1.22	a	0.87	a	*	2.08	a	1.23	a	*
	*n* = 5		*n* = 5			*n* = 5		*n* = 5		

Significance letters show significant differences (Dunn’s test, p < 0.05) between growth stages, while the asterisks indicate significant differences between the P levels for the water levels and growth stages each (0.05, 0.01, 0.001; *, **, ***).

Under well-watered conditions, panicle formation was positively associated with root biomass, root surface area (SurfArea), and the number of crown roots (Supplement Fig. S9A). In contrast, under WS, root and shoot-related parameters were negatively correlated to panicle biomass. Only higher investment in roots, reflected by a greater root-to-shoot ratio showed a positive relationship with panicle formation and panicle P content (Supplement Fig. S9B).

**Fig. 6:**
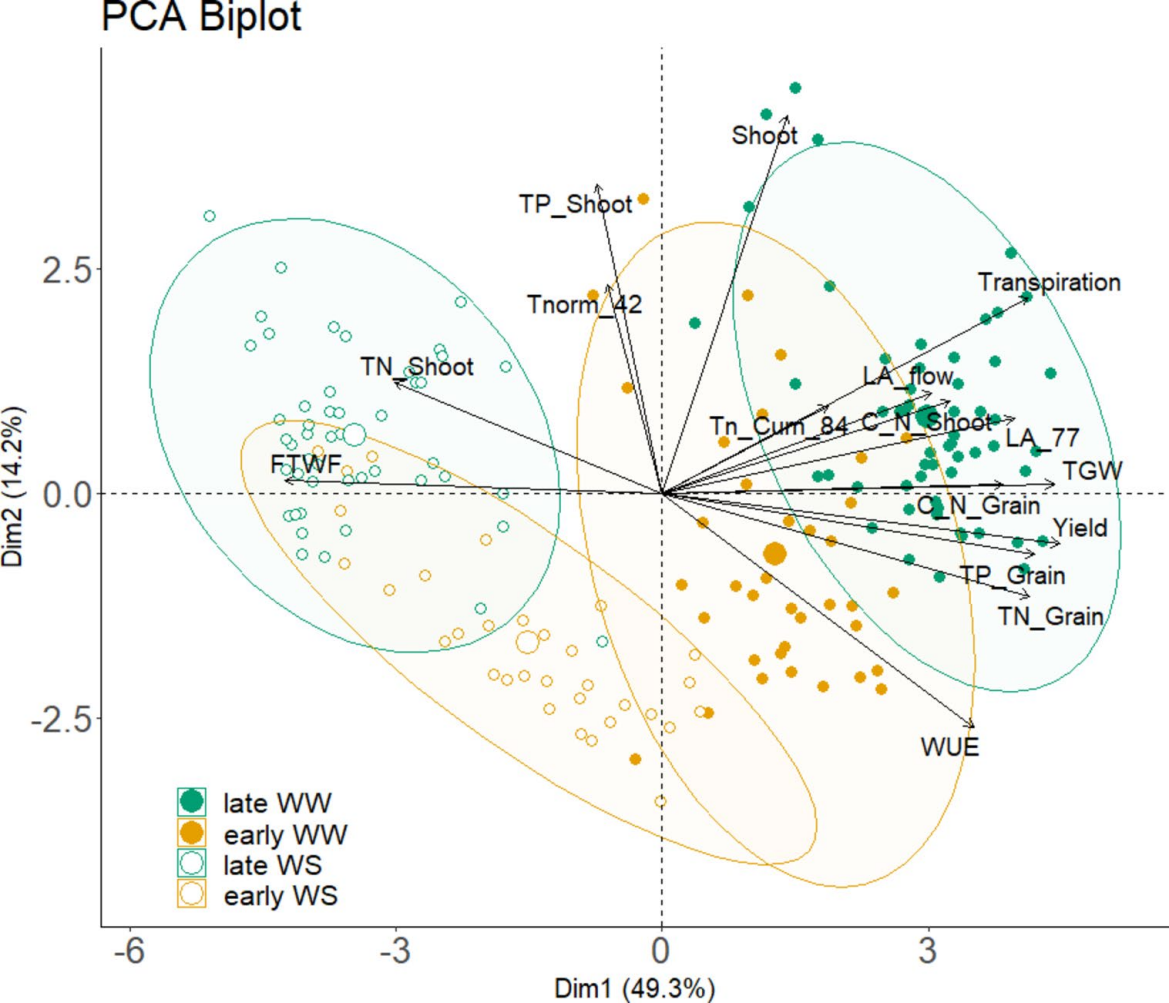
The PCA Biplot shows data variation of the main experiment. The parameter in PCA: C/N ratio of grain (C_N Grain) and shoot (C_N Shoot) biomass, fraction of water transpired before flowering (FTWF), leaf area at flowering (LA_flow), leaf area at DAS 77 (LA_77), shoot biomass (Shoot), thousand-grain weight (TGW), normalized transpiration rate at DAS 42 (Tnorm_42) total nitrogen shoot (TN Shoot), total phosphorus shoot (TP Shoot), total nitrogen grain content (TN Grain), total phosphorus grain (TP Grain), total amount of transpired water (Transpiration). The larger circles show the cluster center of each cluster, while the smaller circles show individual data points.

Regarding plant water consumption, the PCA biplot (Fig. 6) revealed a negative interaction between the FTWF and yield formation, thousand-grain weight (TGW), grain and shoot C/N ratios, as well as grain P and N contents. These parameters, along with transpiration and leaf area at DAS 77, had the highest impact on the first PCA dimension, explaining 49.3% of the data variance. The second dimension, accounting for 14.2% of the variance, was influenced by shoot, shoot total P content (TP Shoot), normalized transpiration at DAS 42 (T_norm_ 42), WUE and transpiration.

The PCA analysis revealed significant clustering of treatments based on water availability and genotype grouping, resulting in distinct combinations: early maturing well-watered, early maturing water shortage, late maturing well-watered and late maturing water shortage.

The late maturing well-watered cluster was clearly separated from the early and late maturing genotypes under water shortage. The early maturing genotype WW cluster was significantly distinct from the late maturing WW cluster. In contrast both early maturing WW and WS overlaped (Fig. 6).

## Discussion

The reported thresholds for sorghum P deficiency depended on the plant development stage during the life cycle. At early stages (23–39 DAS), P concentrations below 2.6 mg g^− 1^ in plant tissues can lead to yield reductions^29^,while at maturity, lower P concentrations around 0.42 mg g^− 1^ have been reported^28^. In our study, sorghum grown in low P soil exhibitedP deficiency during early growth stages, with recorded P concentrations of 2.01 mg g^− 1^. At maturity, P concentrations were 0.51 mg g^− 1^, indicating moderate P limitation, (Table 2). However, yield remained unaffected by soil P level as long as the water supply was sufficient, although shoot biomass was impacted (Figs. 1 and 2). These findings indicate that sorghum possesses unique attributes to compensate for P limitations, including an improved P uptake as the plant develops and a partitioning strategy aimedat preventing yield loss^30^. Sorghum genotypes from West Africa- where soils are highly P deficient- exhibited a greater efficiency in P uptake and utilization compared to genotypes of different origins^31^. This highlights the potential for breeding efforts to develop sorghum varieties with improved P use efficienciy^31^.

Strategies to improve nutrient and P acquisition include symbiosis with arbuscular mycorrhizal fungi (AMF), which enhances P uptake under low soil P availability through a P-scavenging approach. However, P-mining strategies, such as increased P solubility via active-release of organic acids, have been proven more effective than mycorrhization under severely P-deficient soil conditions^32^. In our study, the contribution of AMF to nutrient acquisition varied over time and with P supply. At anthesis, and only under low P, there was a significant and positive correlation between the panicle P and N content and the degree of AMF infection rates (Supplement, Fig. S8B). Additionally, early maturing genotypes from West Africa exhibited a significant increase in AMF infection rates at low compared to high P levels at anthesis (Table 2). The results suggest that the random AMF infection rates alone may not be areliable proxy to estimate nutrient supply to the host plant. Accurate estimationwould require cross-validation using isotope tracers or reference treatments with soil sterilization, given the dynamic and regulated nature of the the plant- AMF interaction, where both partners actively influence nutrient exchange^33^. Apart from mycorrhization, low P availability may have induced different P mobilizing processes, such as carboxylate release^32^ and enhanced phosphatase activity^34^. Thus, soil P availability and P uptake may have increased despite similar AMF infection rates.

Under P deficiency, plants can adjust their carbon partitioning by increasing their root-to-shoot ratio^35,36^. This was observed for the early maturing genotypes during stem elongation and maturity (Supplement, Table S7). However, at anthesis, the root-to-shoot ratio remained high at both P levels. The late maturing genotypes showed only a trend of a higher root-to-shoot ratios at maturity between the P levels (Supplement, Table S7). In contrast, the genetic adaptation of the early-maturing genotypes to the nutrient-poor soils of sub-Saharan Africa leads to increased root-to-shoot ratios to cover the high nutrient demand.

A consequence of P limitation was a prolonged plant development of all sorghum genotypes, including delayed LA development (Supplement Fig. S5 & S6) and flowering (Fig. 5), consistent with observations under field conditions^28^. Organic residues improved P availability under low P conditions, as evidenced by a significant increase in LA at DAS 48 (Supplement Fig. S6). Additionally, the flowering time point was significantly shortened. In contrast, organic residues had no effect on the LA or flowering time point in the high P soil (Fig. 5). Several studies revealed that cowpeas can mobilize P from non-labile sources and enhance the P cycling^37,38^, making P more accessible to subsequent crops^27^. Field experiments with legume-cereal crop rotations have reportedincreased biomass and yield for cereals (maize and sorghum) compared tocontinuous cereal cropping with little or no mineral fertilizer application^6,27,39^. In this study, however, only a positive trend in biomass increase (Supplement Fig. S1) was observed following legume cropping under WW conditions. Most of the pre-crop biomass, including a substantial portion of the fixed N and mobilized P from the legume, was harvested and removed. However, the subsequent sorghum plants still had a significant advantage, particularly during the early stages of development (Fig. 5 and Supplement Fig. S5 & S6).

Besides mere productivity, fertilizer costs significantly affect the farmer’s revenues, which can increase by reducing fertilizer consumption in legume-cereal crop rotations^40^. This study demonstrated that sorghum genotypes could maintain yield production under low P soil contents due to their P mobilizing strategies. We could not prove that increasing P uptake by soil exploitation strategies was linked to larger root systems^41^, which might have been an artifact of the experimental setup. Most probably, P mobilization processes associated with AMF symbiosis, enhanced at essential time points of plant development, were the main belowground strategies for the plant P uptake. Simultaneously, the high plasticity of the root-to-shoot ratio was a critical trait ensuring P supply to the sink organs within the plant and sustained yield production under low P soil levels.

Drought is a major constraint for crop yield production. Although sorghum is considered a drought-adapted crop, there is a large genetic variability^20^. In terms of yield production, the early maturing genotypes outperformed the late maturing ones. Although water shortage caused significant yield reduction across all genotypes by (Fig. 2). The results indicate that the early flowering of the genotypes Lata 3 and Grinkan resulted in the advantage of higher water availability after flowering compared to the late maturing genotypes. The observation aligns with findings from other terminal drought studies on drought-adapted sorghum genotypes, which are characterized by early development and smaller plant sizes^42^. Efficient transpiration distribution is crucial for yield maximization with a threshold of 30% of total water transpired occurring after flowering to enhance yield potential^43,44^.This is in accordance with the results of our study (Fig. 3). For the early maturing genotypes Lata 3 and Grinkan, transpiration after flowering accounted for an average of 25–47% of total water use (Supplement Fig. S3), enabling them to achieve the highest yield production under drought conditions. The late maturing genotypes had less than 20% of the available water left for transpiration after flowering, resultingin negligible yield production. The harvest index for the early maturing genotypes increased from 10% with 10% water used after anthesis to 45% when 20–30% of water was used post anthesis. Additionally, a low FTWF was positively correlated with higher yields (Fig. 3).

However, the water conservation strategies differed between the early maturing genotypes due to their morphological and physiological adaptations. Lata 3 maintained a relatively high transpiration rate per unit leaf area, mainly caused by a lower LA (Supplement: Fig. S5), a trait also confirmed by other studies^45^. In contrast, Grinkan exhibited water conservation characteristics even under well-watered conditions, as T_n_ was reduced compared to the other genotypes across most of the treatment combinations (Fig. 4). One possible explanation may be the increasing root-to-shoot ratio evolving first during stem elongation and continuing until flowering. This adaptation enhances water availability by increasing root water influx^46^. In contrast, drought adaptation through reduced transpiration per leaf area would result in lower photosynthetic activity, making such strategies more suitable for environments with low but frequent precipitation events^47,48^. Altered physiological changes in aboveground plant organs in response to drought stress are often associated with an altered carbon allocation to belowground traits^49^. This was evident in our dataset, as the early maturing, water conserving varieties showed a relatively higher investment in root biomass (Table 2). High plasticity of water conversation traits in sorghum genotypes may be crucial for improving yields in water-prone environments^50^. Key traits of early maturing genotypes, such as rapid development coupled with a higher root-to-shoot ratio, increase potential water uptake relative to LA andenable sustained yield production under drought conditions compared to the late maturing genotypes. The advantage is further enhanced by incorporating water-saving strategies, such as low Tn, as demonstrated by Grinkan.

The results revealed that, on average, Sorghum could maintain good productivity even on P-limited soils. However, both shoot biomass and yield significantly declined under water shortage compared to plants grown in well-watered, high P soil conditions. These results highlight the critical role of soil water content in controlling plant P supply through P mobilization and diffusion processes in the soil^51^. Due to the high reactivity of P, only a low concentration-ranging from 0.6 to 11 µM-is available in the soil solution^52^. The transport of these P ions to the plant is exacerbated under restricted soil water contents, as it is mainly driven by diffusion^53^.

Early maturing genotypes outperformed late maturing ones under WS conditions, not exclusively due to their faster development but mainly due to their water-saving traits, resulting in higher yields (Fig. 4). Remaining organic residues of the cowpea pre-cropping accelerated plant development by increasing plant available P and possibly other nutrients, resulting in a delayed flowering time in the P deficient soil (Fig. 5). However, this faster development under low P with organic residues was accompanied by a higher LA during early growth stages (Supplement, Fig. S5) and higher pre-flowering transpiration rates (Fig. 3). Cosequently, under water shortage, less than 30% of transpiration occurred post-flowering, causing yield reductions^43,44^. The advantage of faster development was thus leveled out by increased pre-flowering water use.

To implement sustainable management systems, particularly in drought-prone areas, careful selection of pre-crops is crucial to ensuring nutrient input into low-fertile soils and maintaining adequate water supply for main crops^54^. The knowledge about the soil rhizosphere interactions and the influence of the root morphology on yield production in semi-arid regions may be as relevant^55^, as evidenced by this study. An increased root-to-shoot ratio emerged as a key plant trait, crucial for coping with both P deficiency^36^ and water shortage^46^, irrespective of the plant development stage. Our results also indicate that sorghum genotypes with a higher root-to-shoot ratio exhibit enhanced water-saving traits at anthesis, contributing to greater yield production at maturity (Supplement Fig. S6b and S6c).

Tube density was selected to mimic plant density under field conditions. Nevertheless, cultivation in tubes restricts lateral root growth and eliminates root-root interaction among individual plants. Of higher relevance for this study was the limitation of soil depth to approximately one meter. Although 1.2 m is relatively deep, highly weathered tropical soils often exceed this soil depth, and Sorghum which is well adapted to such soils, is reported to reach rooting depths of 1.5 m^56^ to 1.85 m^57^. These deep-rooting crops can access additional water resources, sustaining growth and yield production by utilizing moisture stored in deep sub-soils^55^.

This trait may be particularly relevantfor the late maturing genotypes, which typically produce higher total shoot and root biomass. For instance, M35-1 successfully utilized in ICRISATS’ breeding programs, washighly regarded bylocal farmers for its performance^58^. The restricted soil volume, specifically the limited tubes depth, likely constrained the late-maturing genotypes’ ability to fully express their potential for drought resistance in this study.

## Conclusions

Sorghum genotypes displayed a high potential for sufficient P mobilization and P uptake, achieving reasonable yield under well-watered conditions, even on infertile soils. In contrast, water scarcity severely restricted P uptake and yield. Consequently, water-saving traits and the optimal timingof water consumption were essential for sustaining yield production. Morphological and physiological changes revealed the close interactions between above- and below-ground traits and their functions under multiple resource limitations. For nutrient-poor soils in semi-arid regions with high drought risk, the most effective combination of traits appeared to be early-maturing varieties with high root-to-shoot ratio, rapid AMF establishment, and low T_n_. In this study, this combination was exemplified by genotype Grinkan. Such genotypes exhibit robust P uptake withstanding low soil availabilities, conserve water before flowering, and reduce their post-flowering water stress, making them well-suited for challenging environmental conditions.

## Materials and methods

### Experimental set up

**Fig. 7:**
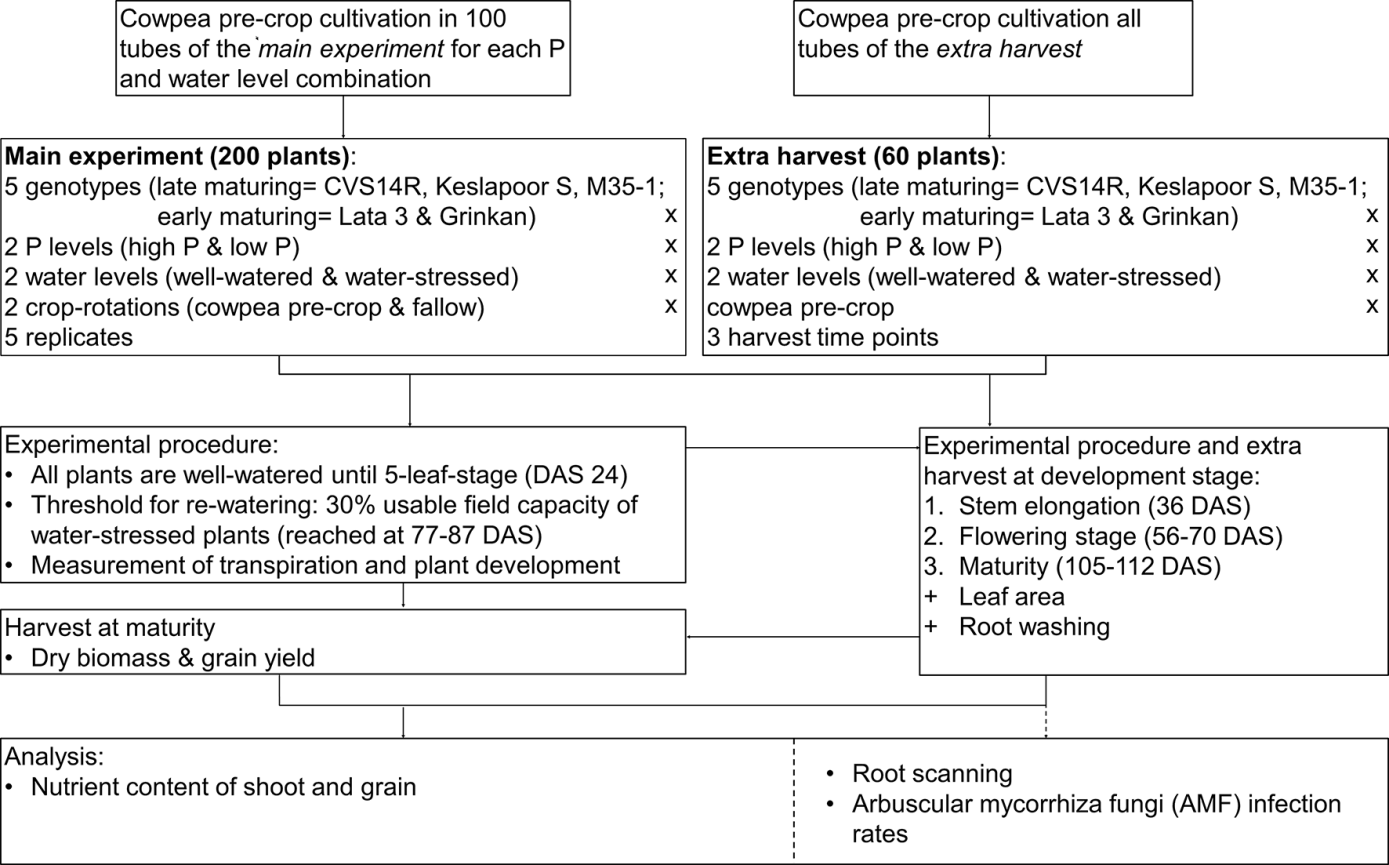
Flowchart of the experimental procedure.

The experiment was conducted at the Lysi-Field facility of the International Crop Research Institute for the Semi-Arid Tropics (ICRISAT India, 17°31’03.6"N and 78°16’36.0"E). The study outline is described in Fig. 7. A detailed description of the study area, facility setup, and soil filling is provided by several studies^59–61^. Briefly, semi-field conditions were created by placing cylindrical PVC tubes (height 120 cm, diameter 25 cm) in a trench. Thus, a planting density of approximately 16 plants m^− 2^ was reached. A mobile rain-out shelter allowed the experiment to be conducted without uncontrolled wetting events during rainfall. A factorial design with four major treatments (low P and high P combined with well-watered (WW) and water-stressed (WS) conditions) across two types of land use (cowpea pre-crop and fallow) and five sorghum [*Sorghum bicolor (L.) Moench*] genotypes was conducted in this study. The water content of each tube was maintained at approximately 80% plant-available water-holding capacity (paWHC) for the WW treatment, while for the WS treatment, the soil in each tube was allowed to dry down to 30% paWHC before water was added. Water was applied as surface irrigation. Five replicates per treatment combination resulted in a total of 200 tubes, i.e., sorghum plants, referred to as the main experiment. In order to get affordable information about belowground sorghum root traits, an additional set of smaller tubes was used, with the same height (120 cm) as in the main experiment but with a smaller diameter of 16 cm. The set of smaller tubes will be addressed as *extra harvest* in the following text. Destructive sampling of the extra harvest was conducted at stem elongation (36 DAS), anthesis (56–70 DAS), and maturity (105–112 DAS). This subset of tubes for destructive harvest was grown solely on soils where pre-crops were previously cultivated and were exposed to both water and P levels. In total, additional 60 tubes, located in the same trench, were used for root parameterization.

### Soil parameters

Soil originating from the ICRISAT station was used to fill the tubes with a bulk density of 1.4 g cm^− 3^^59^. The soil texture consisted of 17% silt, 21% clay, and 62% sand and was classified as a loamy sand Alfisol. Plant available volumetric water content was 25.71%, which was used to calculate thresholds for WW and WS treatments. The Alfisol exhibited Olsen P levels of 2.57 mg P kg^− 1^ and 32.98 mg P kg^− 1^ for the low and high P treatment, respectively. Organic C and total N contents were 0.44, 0.544% and 0.04, 0.51% for high and low P, respectively, and were determined by an elemental analyzer. The pH (H_2_O) values differed between high P and low P soil with 5.5 and 6.1, respectively.

### Plant cultivation

The experiment, including the entire crop rotation, was conducted from July 2018 to February 2019. During that period, the maximum and minimum temperatures ranged from 19.2 to 33.6 °C to 5.2–23 °C, respectively. The pre-crop cowpea grew from July till the end of September 2018. Five cowpea seeds (genotype: Giant Russian) were sown, and the plants were thinned out to one plant per cylinder. The plants were irrigated sufficiently throughout the cultivation period, and pesticide and fertilizer application (except for P in the low P treatment) was conducted according to common practice. Sorghum was planted in the post-rainy season from October 2018 until February 2019. In total, five sorghum genotypes were grown, with two early maturing (Lata 3, Grinkan) and three late maturing genotypes (CSV14R, Keslapoor, M35-1). M35-1 is a popular variety grown in the post-rainy season in India because of its stable yield performance under terminal drought conditions^62^. This variety often serves as a control in germplasm studies to identify new accessions or breeding progress of genotypes under unfavorable water and nutrient conditions. It also serves as a genetic source for new breeding cultivars or hybrids^62^,^58^ and, thus, was used as a control in our study.

Six sorghum seeds of each genotype were sown two weeks after the cowpea harvest. Additional sorghum plants were grown in pots surrounding the trench to minimize border effects. Pest control was applied according to common practice. In addition, carbofuran (2,2-Dimethyl-2,3-dihydro-1-benzofuran-7-yl methylcarbamate) was regularly sprayed against the fall armyworm (*Spodoptera frugiperda)* and successfully prevented biomass loss. Fertilizer was applied according to soil P treatment before sowing as topsoil application. Low P main experiment tubes received 2.3 g (low P extra harvest tubes 1.4 g) urea, and high P main experiment tubes 5 g (high P extra harvest tubes 3.1 g) di-ammonium phosphate (DAP). At 25 DAS, the 5-leaf stage, plants were thinned to one plant per cylinder, followed by soil saturation at DAS 27. Afterwards, plastic sheets and a layer of 2 cm polyethylene beads were placed on the soil surface to prevent about 90% of soil evaporation^48,59^. The initial weight for the field capacity calculation per cylinder was recorded when no more leaching from the bottom of the tubes was detected. The first weight was the reference for further water content and transpiration calculations^59^. Weekly weighing ensured the controlled water content monitoring, and water was added in order to maintain the water levels of 80% or 30% paWHC. Sorghum was harvested after reaching physiological maturity. The individual plants were separated into leaf, stem, panicle, tiller, and tiller panicles, oven-dried at 60 °C and weighed. After threshing, grain weight and thousand-grain weight (TGW) were recorded. The samples were milled separately at the Department for Crop Physiology lab facilities at ICRISAT, and subsamples were sent to the University of Goettingen for further lab analyses.

Destructive sampling of the extra harvest was conducted to get above- and belowground biomass data. Although the biomass production in the extra harvest tubes was reduced to around 24% compared to the larger tubes, we assume that other morphological traits (such as root biomass) changed proportional to the plant size and that physiological traits were unaffected (see section *Phenological development and resource acquisition*). Furthermore, the flowering time point (DAS 54–71), physiological maturity (DAS 105–112), and genotype-specific patterns in development and biomass accumulation were similar for the sorghum plants in the tubes for destructive harvest, comparable to the plants in the main experiment.

The main experiment results should provide detailed information about the treatment effects on morphological and physiological traits of the sorghum genotypes. At the same time the extra harvest should link the above and belowground interactions to the quantified parameters.

Harvest time points of the extra harvest were selected based on the plants’ development status (stem elongation, anthesis, and maturity), while the sample preparation was equal to the procedure mentioned above. In addition, root biomass was washed, collected with sieves, and stored in 70% ethanol until root scanning was completed. A small subsample was immediately frozen at -20 °C for arbuscular mycorrhiza infection rate estimation. After crown root counting, the roots were oven-dried and milled.

### Field measurements

Sorghum plant development was recorded bi-weekly with the following parameters: plant height until the last developed leaf, number of developed leaves, number of developing leaves, number of senesced leaves as well as tiller numbers and height. A developed leaf was counted when the lamina was fully unfolded at the stem. Senesced leaves were characterized by over 50% dried out, yellowish, or dead appearance. Leaf area was measured from all green, developed leaves from the fifth leave upwards of all plants, including tillers. The leaf area of the first four leaves was negligible and was therefore not measured. Total leaf length was measured from the stem’s separation point until the leaf’s tip and the width at the widest point of the leaf. Calculation of leaf area was obtained by multiplying the product of leaf length and width with the shape correction factor 0.75^63^. After the emergence of the first flower, the flowering date was recorded daily and determined when more than 50% of the panicle was covered with anthers. Flowers were covered with light nylon bags to prevent damage by animals.

### Analysis and calculations

For further analysis, the cowpea and sorghum plant samples were dried at 60 °C and ground to a fine powder in a ball mill (MM200 Retsch Haan Germany). Three to five mg of the plant samples were weighed into tin capsules (IVA, Meerbusch, Germany) to determine N and C contents at the elemental analyzer (Flash, ThermoFisher Scientific, Bremen, Germany). The analyses were conducted at the Centre for Stable Isotope Research and Analysis (Georg August-University Goettingen).

Total N (TN) content was calculated by multiplying the N fraction by the biomass (g) of the plant parts, resulting in total biomass N content. Total P content was extracted by the nitric acid pressure digestion method^64^ and was measured on an inductively coupled plasma optical emission spectrometer (ICP-OES, Thermo Scientific iCap 6000 Series).

The weekly transpiration (L week^− 1^) was estimated by calculating the weight difference between the weights of two successive weeks plus the added irrigation water^59^. Consequently, the total transpiration was the sum of all weekly transpiration values from initial weighing until harvest. The quantification of the normalized transpiration rate per unit of leaf area (T_n_) allows an easy and nondestructive assessment of the sorghum varieties’ transpiration characteristics throughout the plant development. T_n_ is defined as unit transpiration (L week^− 1^) per unit of leaf area (m^2^)^17^.

Another parameter estimating the plant’s water use pattern during growth is the fraction of transpired water till flowering (FTWF). FTWF is calculated by dividing the cumulative transpiration (L plant^− 1^) until flowering by the total transpiration (L plant^− 1^) until harvest.

Harvest index (HI) in % was calculated by dividing grain DM (g) by shoot biomass DM (g) multiplied by 100^65^.

The water use efficiency (WUE) is defined as the unit grain DM (g) per unit water transpired (L)^66^.

Root colonization with arbuscular mycorrhiza fungi (AMF) was determined by the modified root staining method^67^. Roots with approximately one cm length were heated in 10% KOH, stained with a 5%-ink-vinegar solution, and stored in glycerol at 4 °C until AMF was counted. AMF colonialization was determined by a modified root segment ± method^68^ with the AMF colonization per root area. Therefore, a light microscope (Olympus BX40 with Olympus CMOS-camera SC50M) with a magnification of 150x (Olympus Ach 10x/0.25 ph ∞ 0.17, ocular 15x, Olympus Europe SE & Co. KG, Hamburg, Germany) in 4.9 Mpx resolution was used. AMF structures were visible without a filter, and pictures were saved as TIFF with JPEG compression. Further counting was conducted by the software tool ‘Fungi Tagger’ (https://gitlab.gwdg.de/sstock1/fungi_tagger)^69^. The remaining washed roots were scanned with a flatbed scanner, and the image-analyzing software WinRHIZO 2013e (Regent Instruments Inc., Québec, Canada) was used for root trait analysis, which where root length (Rlength) (cm), root surface area (SurfArea) (cm^2^), and specific root density (SRD, cm g^− 1^), with SRD being calculated by dividing Rlength (cm) by the root mass (g).

### Statistical analysis

Data preparation and outlier elimination were performed using Microsoft.Excel (Version 16.29.1). Statistical analysis and graphical visualization were conducted using R.Studio (Version 4.2.2). Shapiro-Wilk test and Levene’s test were performed to check data for normal distribution and homogenous variances, respectively. If necessary, data were log-transformed to fulfill the assumptions, and an analysis of variance (ANOVA) was performed, followed by Tukey post hoc tests. The Kruskal-Wallis test was carried out for non-parametric data, followed by Dunn’s post hoc test to identify statistical differences between groups. Significant differences were detected at a threshold of *p* < 0.05. The corrplot function of R was used to perform Spearman’s rank correlation coefficient test with a threshold of *p* < 0.05.

## Electronic supplementary material

Below is the link to the electronic supplementary material.

Supplementary Material 1

## Data Availability

The data will be made available from the corresponding author on request.
