# Supplementary material for: Distinct water and phosphorus extraction patterns are key to maintaining the productivity of sorghum under drought and limited soil resources
Source: Sci Rep. 2025 Feb 10;15:4949. doi: 10.1038/s41598-025-88705-x (PMC11811011; doi:10.1038/s41598-025-88705-x)
Supplement: Supplementary file 1 — Supplementary Material 1 [file 41598_2025_88705_MOESM1_ESM.docx]

# Supplement: Distinct water and phosphorus extraction patterns are key to maintaining the productivity of Sorghum under drought and limited soil resources


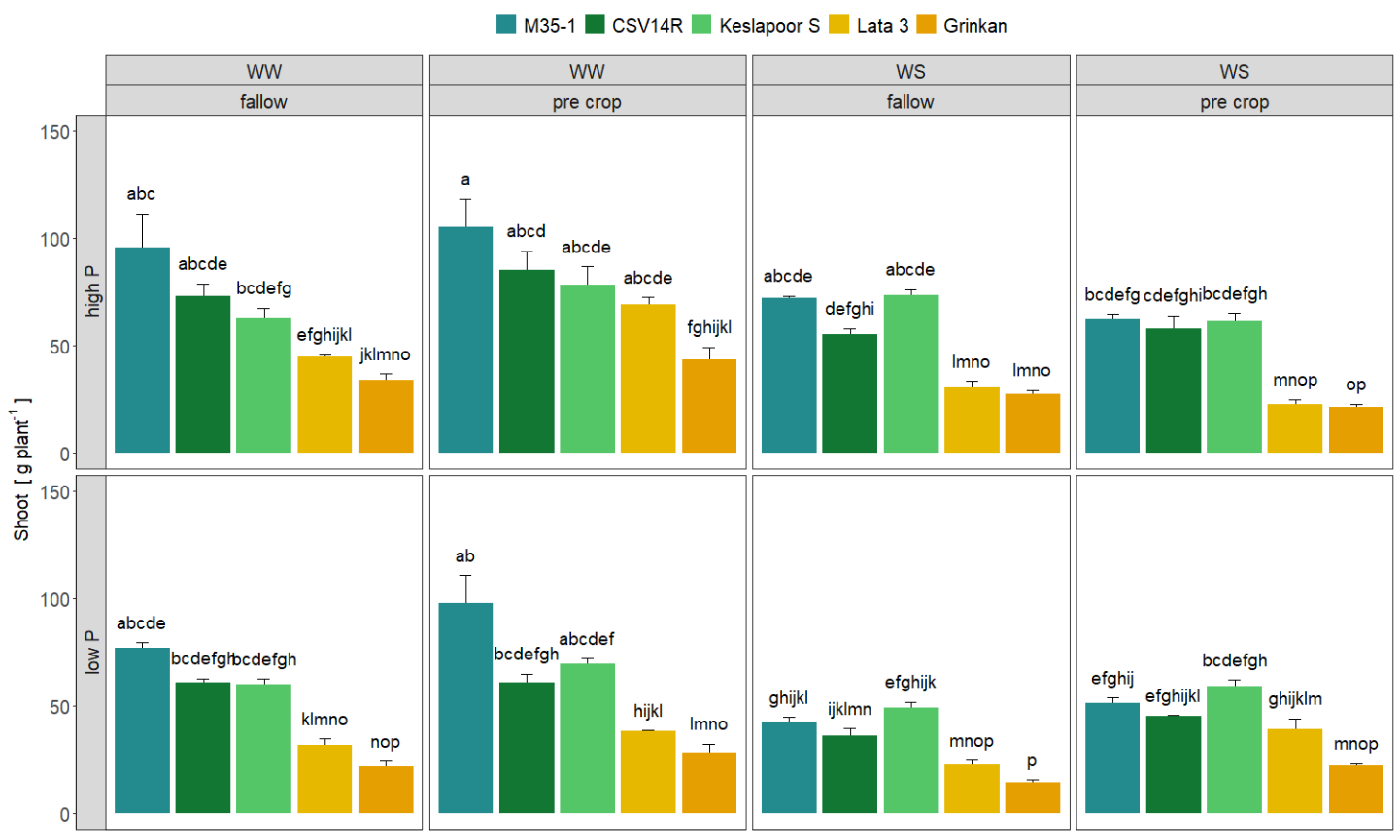


Fig. S1 Shoot biomass of five sorghum genotypes grown under two water levels well-watered (WW) and water-stressed (WS) conditions, two P levels, high and low P and with fallow and pre-crop 15N sources. Late maturing genotypes are represented by CSV14R, Keslapoor S and M35-1 and early maturing genotypes are represented by Lata 3 and Grinkan. Data show arithmetic means (n=3-5) and standard error (SE) of the mean. The different letters indicate significant difference (p<0.05, Tukey post-hoc test) of log-transformed data.


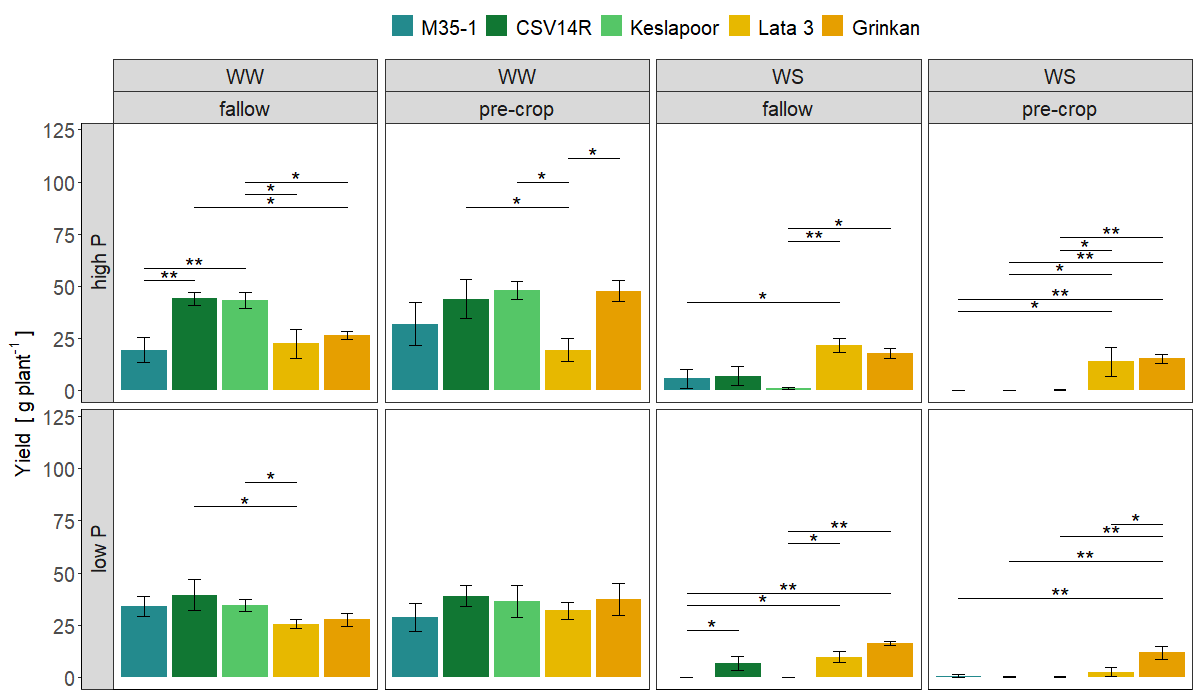


Fig. S2 Yield of five sorghum genotypes grown under two water levels well-watered (WW) and water-stressed (WS) conditions, two P levels, high and low P and with fallow and pre-crop 15N sources. Late maturing genotypes are represented by CSV14R, Keslapoor S and M35-1 and early maturing genotypes are represented by Lata 3 and Grinkan. Data show arithmetic means (n=3-5) and standard error (SE) of the mean. Asterisks show significant differences between the genotypes (p= 0.05, Dunn’s test) with the factor genotype as pairwise comparison.


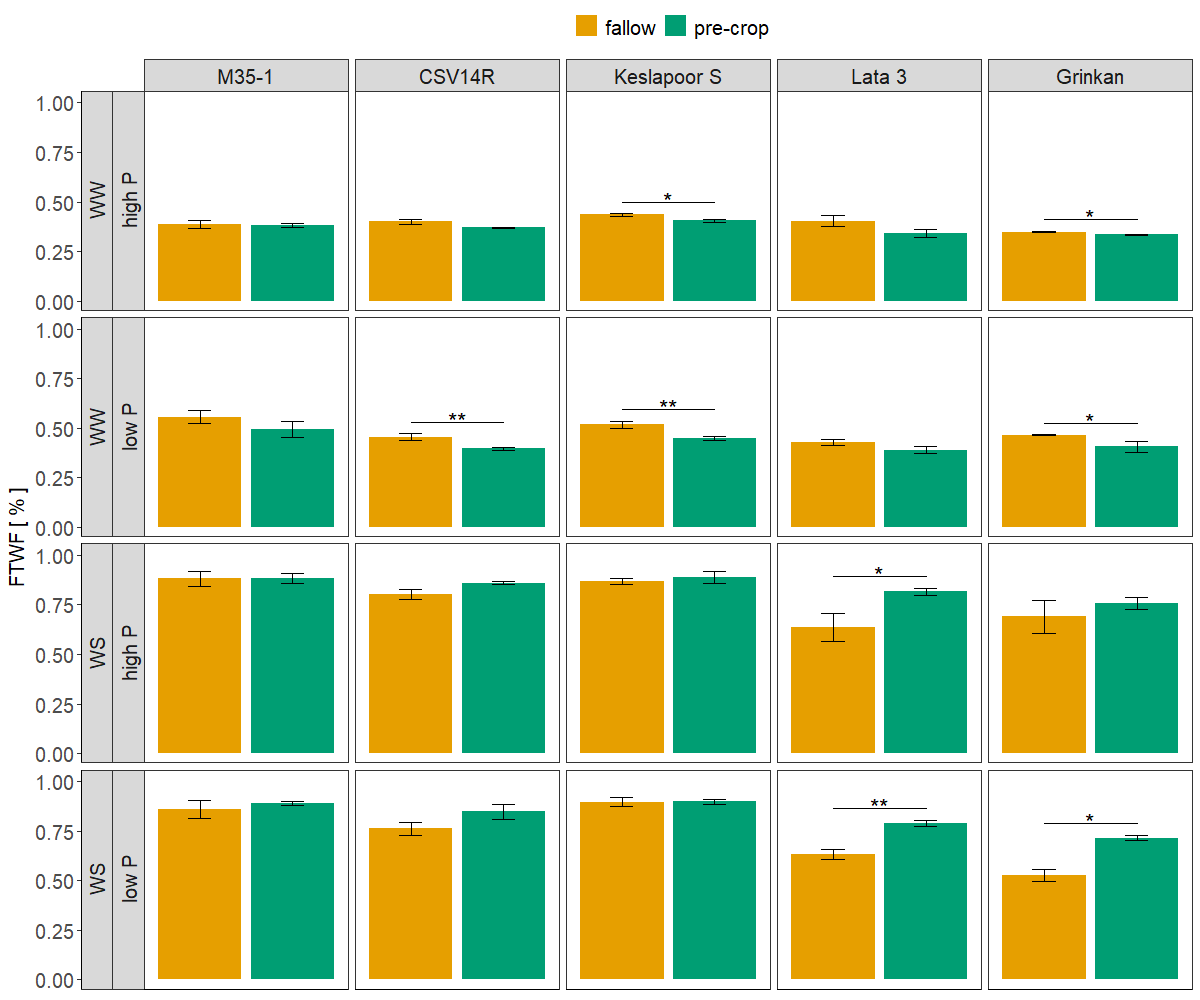


Fig. S3 Fraction of transpired water before flowering (FTWF) in (%) of five sorghum genotypes grown under two water levels, well-watered (WW) and water-stressed (WS) conditions, two P levels, high and low P and with different crop rotations (fallow and pre-crop). Late maturing genotypes are represented by CSV14R, Keslapoor S and M35-1 and early maturing genotypes are represented by Lata 3 and Grinkan. Data show arithmetic means (n=3-5) and standard error (SE) of the mean. Asterisks show significant differences between the crop rotatiom (p= 0.05, Dunn’s test) as pairwise comparison


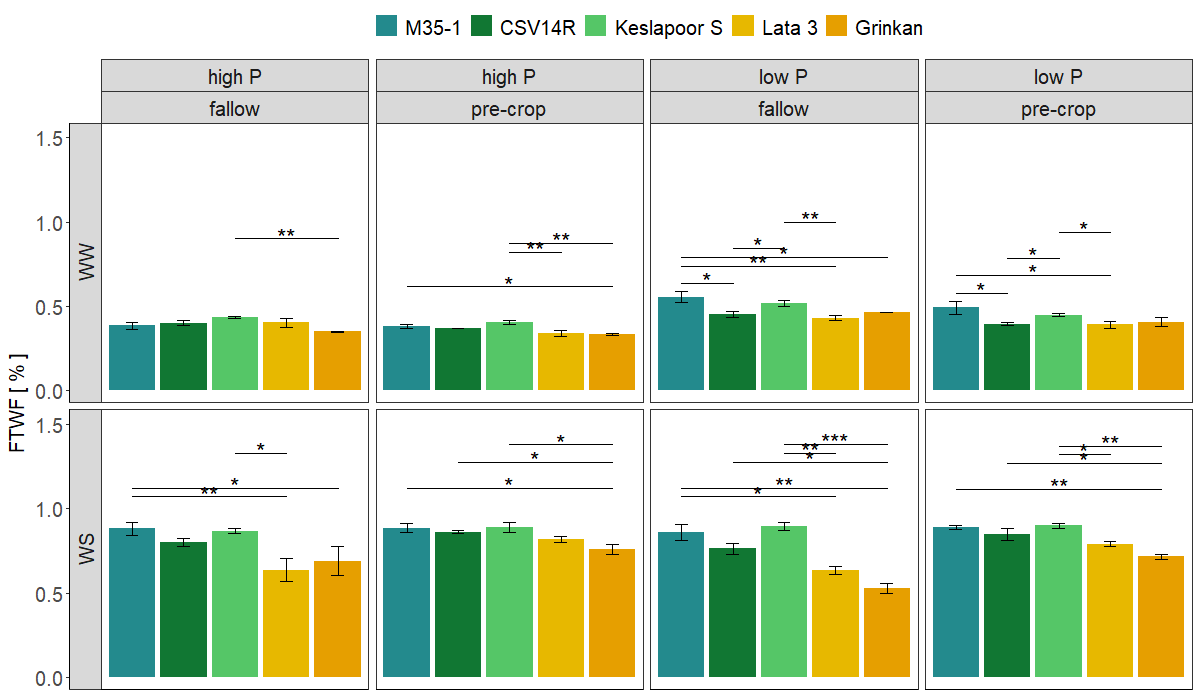


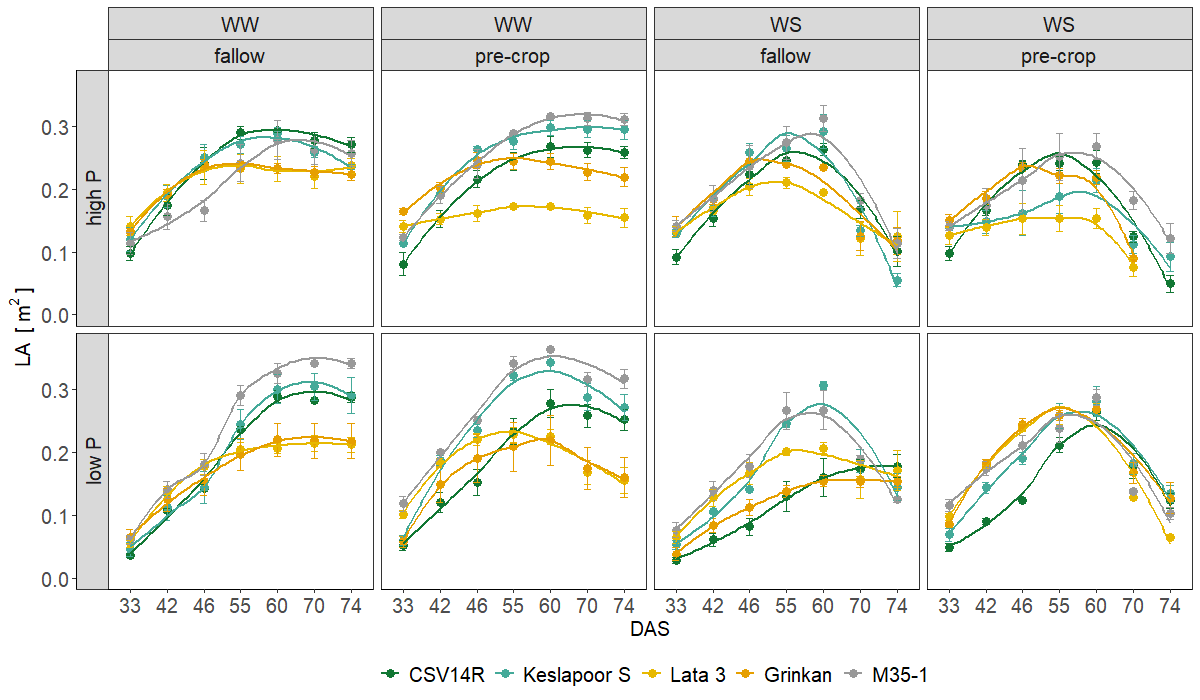
Fig. S4 Fraction of transpired water before flowering (FTWF) in (%) of five sorghum genotypes grown under two water levels, well-watered (WW) and water-stressed (WS) conditions, two P levels, high and low P and with different crop rotations (fallow and pre-crop). Late maturing genotypes are represented by CSV14R, Keslapoor S and M35-1 and early maturing genotypes are represented by Lata 3 and Grinkan. Data show arithmetic means (n=3-5) and standard error (SE) of the mean. Asterisks show significant differences between the genotypes (p= 0.05, Dunn’s test) as pairwise comparison.

Fig. S5 Leaf area (LA) ) of five sorghum genotypes grown under two water levels, well-watered (WW) and water-stressed (WS) conditions, two P levels, high and low P and with mineral and organic 15N sources. Late maturing genotypes are represented by CSV14R, Keslapoor S and M-35 and early maturing genotypes are represented by Lata 3 and Grinkan.


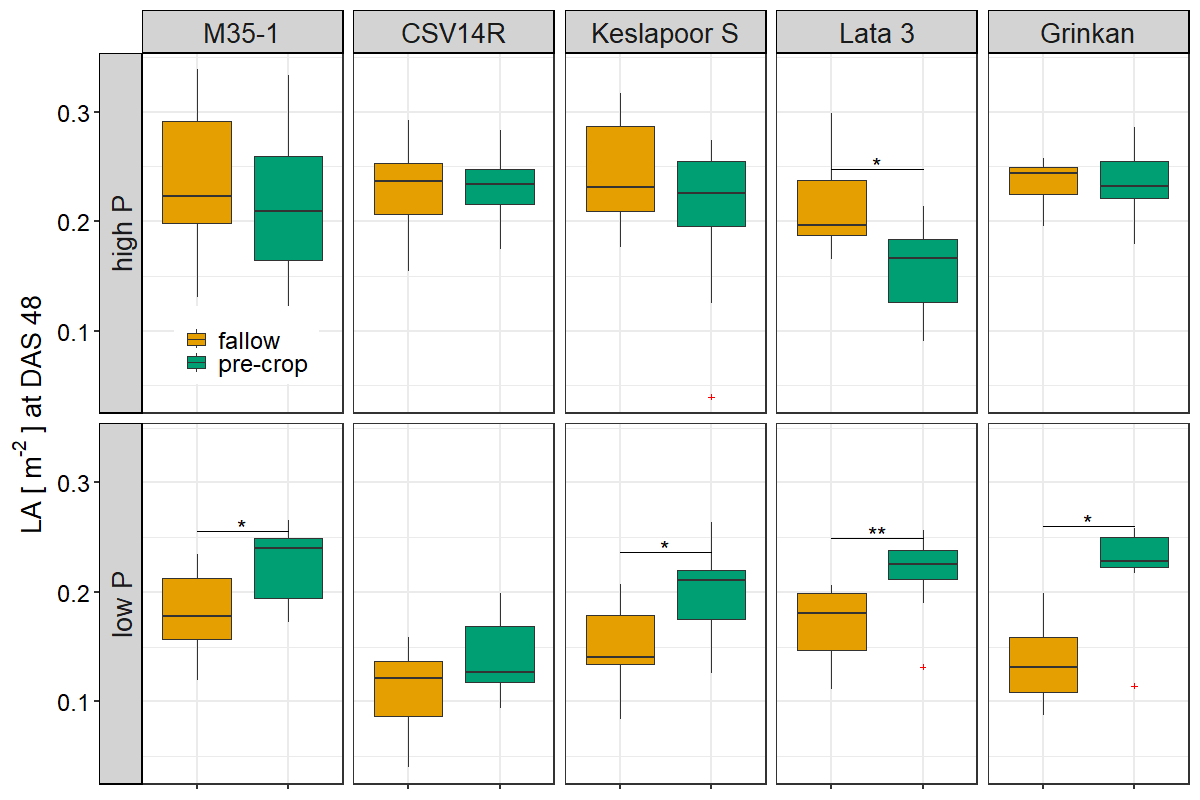


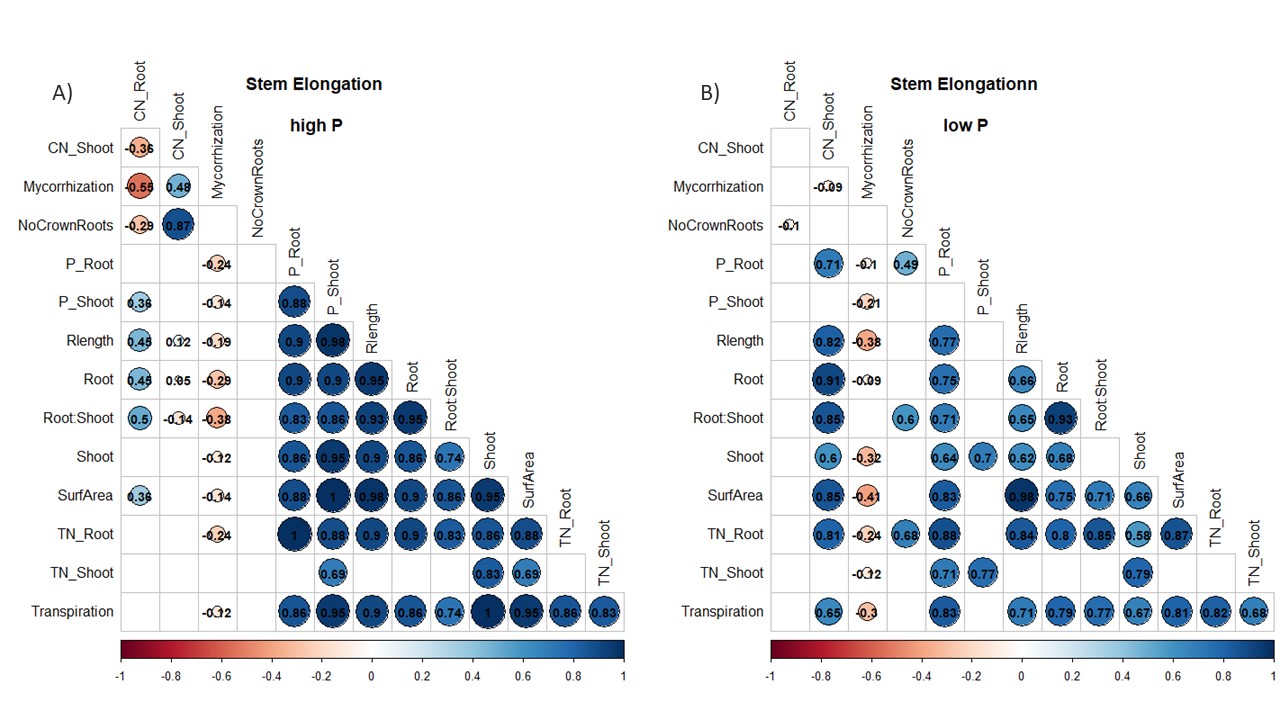
Figure S6 Leaf area (LA) 48 days after sowing (DAS) in m^-2^ of five sorghum genotypes grown under two P levels, high and low P and with fallow (orange) and pre-crop (green). Late maturing genotypes are represented by CSV14R, Keslapoor S and M35 and early maturing genotypes are represented by Lata 3 and Grinkan. At DAS 48 the water supply was still sufficient and shortly before the onset of water limitation.

Figure S7 Correlation matrix between morphological and physiological parameters of five sorghum genotypes. Comparison of additionally harvested plants at stem elongation. Spearman’s Rank correlation test was applied to calculate correlation coefficients. Only significant (p<0.05) results are displayed in the figure and blue and red colors indicate a positive or a negative correlation, respectively. Color intensity and the size of the circles are proportional to the correlation coefficients.

*
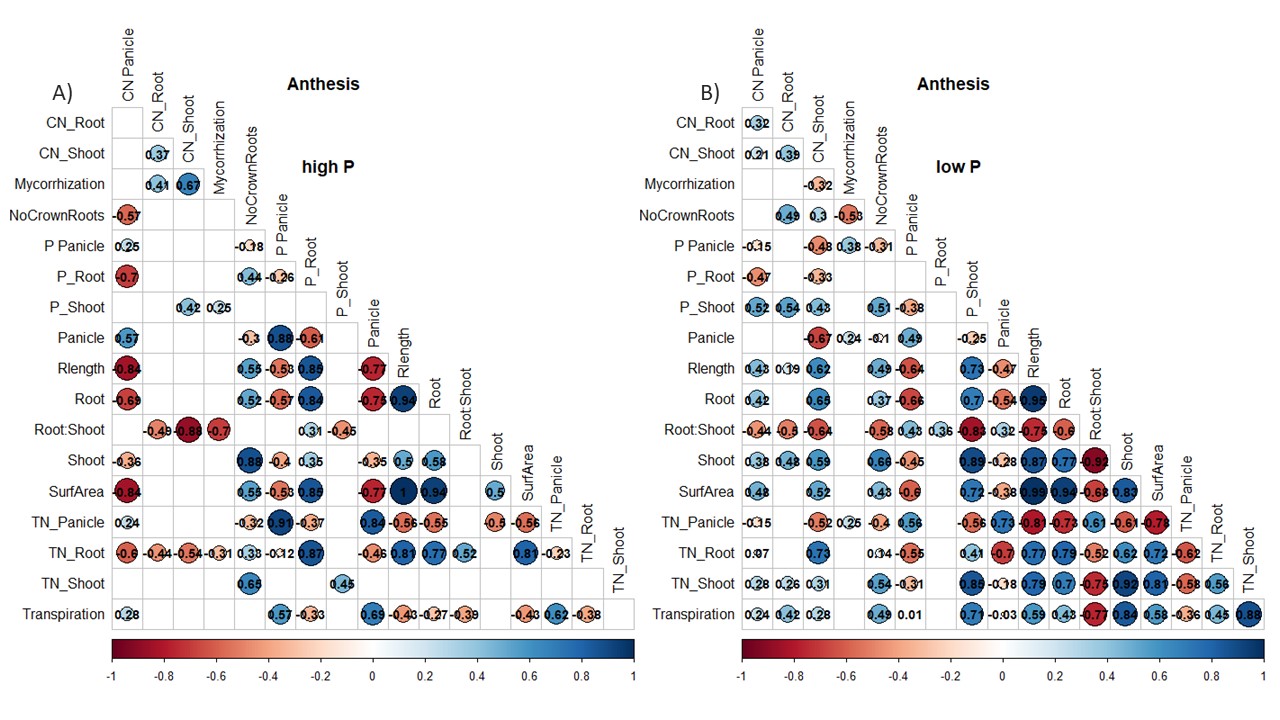
*

Figure S8 Correlation matrix between morphological and physiological parameters of five sorghum genotypes. Comparison of additionally harvested plants at A) high P and B) low P at Anthesis. Spearman’s Rank correlation test (n=20) was applied to calculate correlation coefficients. Only significant (p<0.05) results are displayed in the figure and blue and red colors indicate a positive or a negative correlation, respectively. Color intensity and the size of the circles are proportional to the correlation coefficients.


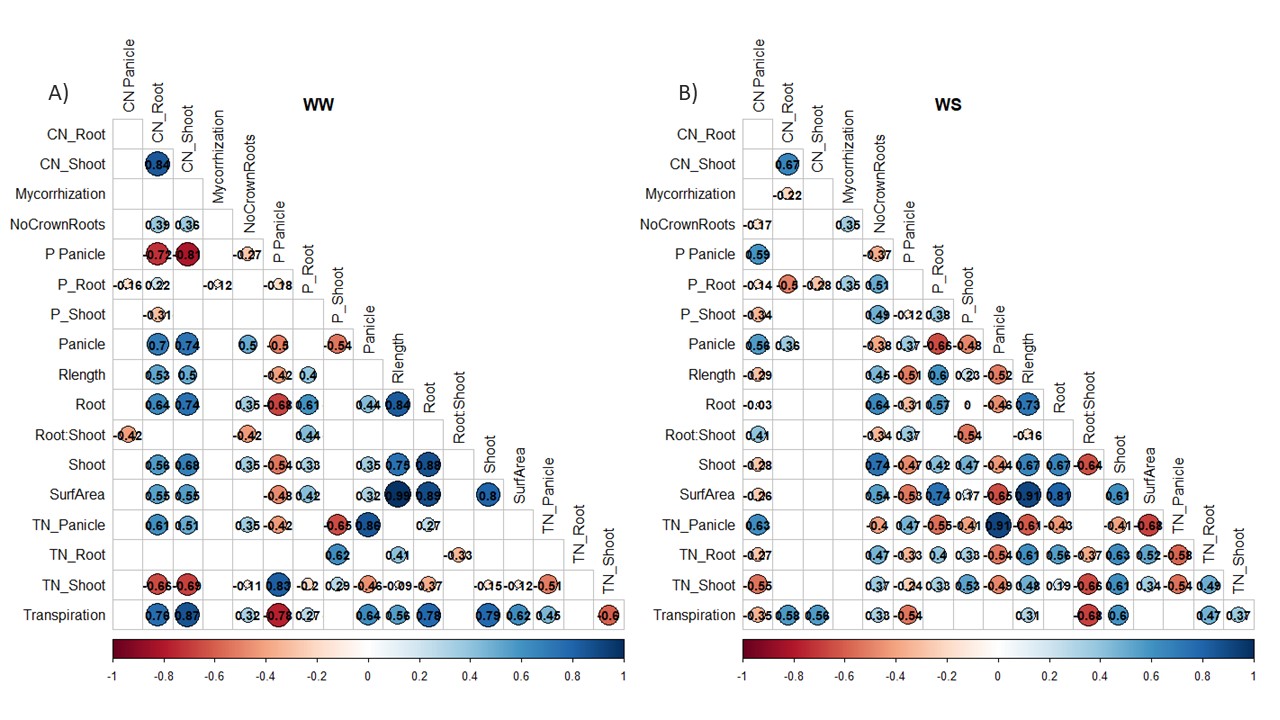


Figure S9 Correlation matrix between morphological and physiological parameters of five sorghum genotypes. Comparison of additionally harvested plants at A) well-watered (WW) and B) water-stressed (WS) combined from the time point of Anthesis and Maturity. Spearman’s Rank correlation test (n=20) was applied to calculate correlation coefficients. Only significant (p<0.05) results are displayed in the figure and blue and red colors indicate a positive or a negative correlation, respectively. Color intensity and the size of the circles are proportional to the correlation coefficients

Table S1: Analysis of variance table of log transformed sorghum biomass data

| Response: log shoot biomass |  |  |  |  |  |  |
| --- | --- | --- | --- | --- | --- | --- |
|  | Df | Sum Sq | Mean Sq | F value | Pr(>F) | Signif |
| Genotype | 4 | 27.0136 | 6.7534 | 138.0598 | < 2.2e-16 | *** |
| P level | 1 | 2.7147 | 2.7147 | 55.4962 | <6.4e-12 | *** |
| Water level | 1 | 6.6832 | 6.6832 | 136.6237 | < 2.2e-16 | *** |
| N source | 1 | 1.2580 | 1.2580 | 25.7179 | <1.1e-06 | *** |
| Genotype : P level | 4 | 0.2194 | 0.0548 | 1.1212 | 0.3486 |  |
| Genotype : Water level | 4 | 0.5912 | 0.1478 | 3.0213 | 0.0197 | * |
| P level : Water level | 1 | 0.0674 | 0.0674 | 1.3772 | 0.2424 |  |
| Genotype : Crop rotation | 4 | 0.2428 | 0.0607 | 1.2407 | 0.2961 |  |
| P level : Crop rotation | 1 | 0.5506 | 0.5506 | 11.2560 | 0.0010 | ** |
| Water level : Crop rotation | 1 | 0.1055 | 0.1055 | 2.1560 | 0.1441 |  |
| Genotype : P level : Water level | 4 | 0.9202 | 0.2300 | 4.7027 | 0.0013 | ** |
| Genotype : P level : Crop rotation | 4 | 0.1058 | 0.0265 | 0.5409 | 0.7059 |  |
| Genotype : Water level : Crop rotation | 4 | 0.0441 | 0.0110 | 0.2251 | 0.9240 |  |
| P level : Water level : Crop rotation | 1 | 0.4209 | 0.4209 | 8.6037 | 0.0039 | ** |
| Genotype : P level : Water level : Crop rotation | 4 | 0.1769 | 0.0442 | 0.9038 | 0.4633 |  |
| Residuals | 153 | 7.4842 | 0.0489 |  |  |  |
| --- |  |  |  |  |  |  |
| Signif. codes: 0 ‘***’ 0.001 ‘**’ 0.01 ‘*’ 0.05 ‘.’ 0.1 ‘ ’ 1 | | | | | |  |

Table S2: Yield. Dunn’s Multiple Comparison between two water levels

| **Genotype** | **Nsource** | **P level** | **.y.** | **group1** | **group2** | **n1** | **n2** | **statistic** | **p** | **p.adj** | **p.adj.signif** |
| --- | --- | --- | --- | --- | --- | --- | --- | --- | --- | --- | --- |
| CSV14R | fallow | high P | Yield | WW | WS | 4 | 5 | -2.45 | 0.014 | 0.0143 | * |
| CSV14R | pre-crop | high P | Yield | WW | WS | 5 | 4 | -2.49 | 0.013 | 0.0127 | * |
| CSV14R | fallow | low P | Yield | WW | WS | 4 | 6 | -2.56 | 0.011 | 0.0105 | * |
| CSV14R | pre-crop | low P | Yield | WW | WS | 5 | 5 | -2.69 | 0.007 | 0.00706 | ** |
| Keslapoor | fallow | high P | Yield | WW | WS | 6 | 5 | -2.74 | 0.006 | 0.00617 | ** |
| Keslapoor | pre-crop | high P | Yield | WW | WS | 4 | 5 | -2.56 | 0.011 | 0.0105 | * |
| Keslapoor | fallow | low P | Yield | WW | WS | 5 | 5 | -2.69 | 0.007 | 0.00706 | ** |
| Keslapoor | pre-crop | low P | Yield | WW | WS | 5 | 5 | -2.69 | 0.007 | 0.00706 | ** |
| Lata 3 | fallow | high P | Yield | WW | WS | 4 | 5 | -0.245 | 0.806 | 0.806 | ns |
| Lata 3 | pre-crop | high P | Yield | WW | WS | 6 | 5 | -0.913 | 0.361 | 0.361 | ns |
| Lata 3 | fallow | low P | Yield | WW | WS | 5 | 5 | -2.61 | 0.009 | 0.00902 | ** |
| Lata 3 | pre-crop | low P | Yield | WW | WS | 5 | 5 | -2.64 | 0.008 | 0.00821 | ** |
| Grinkan | fallow | high P | Yield | WW | WS | 5 | 4 | -1.96 | 0.05 | 0.05 | ns |
| Grinkan | pre-crop | high P | Yield | WW | WS | 5 | 5 | -2.61 | 0.009 | 0.00902 | ** |
| Grinkan | fallow | low P | Yield | WW | WS | 4 | 3 | -2.12 | 0.034 | 0.0339 | * |
| Grinkan | pre-crop | low P | Yield | WW | WS | 3 | 5 | -2.24 | 0.025 | 0.0253 | * |
| M-35 | fallow | high P | Yield | WW | WS | 5 | 5 | -1.57 | 0.116 | 0.116 | ns |
| M-35 | pre-crop | high P | Yield | WW | WS | 5 | 5 | -2.69 | 0.007 | 0.00706 | ** |
| M-35 | fallow | low P | Yield | WW | WS | 5 | 4 | -2.56 | 0.011 | 0.0105 | * |
| M-35 | pre-crop | low P | Yield | WW | WS | 4 | 5 | -2.49 | 0.013 | 0.0127 | * |

Table S3: Dunn’s Multiple Comparison between the two P levels

| **Genotype** | **Nsource** | **Water l.** | **.y.** | **group1** | **group2** | **n1** | **n2** | **statistic** | **p** | **p.adj** | **p.adj.signif** |
| --- | --- | --- | --- | --- | --- | --- | --- | --- | --- | --- | --- |
| CSV14R | fallow | WW | Yield | high P | low P | 4 | 4 | -1.15 | 0.248 | 0.248 | ns |
| CSV14R | pre-crop | WW | Yield | high P | low P | 5 | 5 | -0.731 | 0.465 | 0.465 | ns |
| CSV14R | fallow | WS | Yield | high P | low P | 5 | 6 | -0.365 | 0.715 | 0.715 | ns |
| CSV14R | pre-crop | WS | Yield | high P | low P | 4 | 5 | 0 | 1 | 1 | ns |
| Keslapoor | fallow | WW | Yield | high P | low P | 6 | 5 | -1.46 | 0.144 | 0.144 | ns |
| Keslapoor | pre-crop | WW | Yield | high P | low P | 4 | 5 | -1.22 | 0.221 | 0.221 | ns |
| Keslapoor | fallow | WS | Yield | high P | low P | 5 | 5 | -2.12 | 0.034 | 0.0343 | * |
| Keslapoor | pre-crop | WS | Yield | high P | low P | 5 | 5 | -0.149 | 0.881 | 0.881 | ns |
| Lata 3 | fallow | WW | Yield | high P | low P | 4 | 5 | -0.735 | 0.462 | 0.462 | ns |
| Lata 3 | pre-crop | WW | Yield | high P | low P | 6 | 5 | 1.64 | 0.1 | 0.1 | ns |
| Lata 3 | fallow | WS | Yield | high P | low P | 5 | 5 | -2.4 | 0.016 | 0.0163 | * |
| Lata 3 | pre-crop | WS | Yield | high P | low P | 5 | 5 | -1.51 | 0.131 | 0.131 | ns |
| Grinkan | fallow | WW | Yield | high P | low P | 5 | 4 | 0.245 | 0.806 | 0.806 | ns |
| Grinkan | pre-crop | WW | Yield | high P | low P | 5 | 3 | -0.149 | 0.881 | 0.881 | ns |
| Grinkan | fallow | WS | Yield | high P | low P | 4 | 3 | 0 | 1 | 1 | ns |
| Grinkan | pre-crop | WS | Yield | high P | low P | 5 | 5 | -0.731 | 0.465 | 0.465 | ns |
| M-35 | fallow | WW | Yield | high P | low P | 5 | 5 | 1.78 | 0.076 | 0.0758 | ns |
| M-35 | pre-crop | WW | Yield | high P | low P | 5 | 4 | -0.735 | 0.462 | 0.462 | ns |
| M-35 | fallow | WS | Yield | high P | low P | 5 | 4 | -1.75 | 0.081 | 0.0808 | ns |
| M-35 | pre-crop | WS | Yield | high P | low P | 5 | 5 | 0.643 | 0.521 | 0.521 | ns |

Table S4 Dunn’s Multiple Comparison between the two N sources

| **Genotype** | **Water l.** | **P_level** | **.y.** | **group1** | **group2** | **n1** | **n2** | **statistic** | **p** | **p.adj** | **p.adj.signif** |
| --- | --- | --- | --- | --- | --- | --- | --- | --- | --- | --- | --- |
| CSV14R | WW | high P | Yield | fallow | pre-crop | 4 | 5 | 0.245 | 0.806 | 0.806 | ns |
| CSV14R | WW | low P | Yield | fallow | pre-crop | 4 | 5 | 0 | 1 | 1 | ns |
| CSV14R | WS | high P | Yield | fallow | pre-crop | 5 | 4 | -2.49 | 0.013 | 0.013 | * |
| CSV14R | WS | low P | Yield | fallow | pre-crop | 6 | 5 | -2.11 | 0.035 | 0.035 | * |
| Keslapoor | WW | high P | Yield | fallow | pre-crop | 6 | 4 | 0.64 | 0.522 | 0.522 | ns |
| Keslapoor | WW | low P | Yield | fallow | pre-crop | 5 | 5 | 1.15 | 0.251 | 0.251 | ns |
| Keslapoor | WS | high P | Yield | fallow | pre-crop | 5 | 5 | -1.67 | 0.095 | 0.095 | ns |
| Keslapoor | WS | low P | Yield | fallow | pre-crop | 5 | 5 | 0.149 | 0.881 | 0.881 | ns |
| Lata 3 | WW | high P | Yield | fallow | pre-crop | 4 | 6 | -0.64 | 0.522 | 0.522 | ns |
| Lata 3 | WW | low P | Yield | fallow | pre-crop | 5 | 5 | 1.57 | 0.117 | 0.117 | ns |
| Lata 3 | WS | high P | Yield | fallow | pre-crop | 5 | 5 | -0.94 | 0.347 | 0.347 | ns |
| Lata 3 | WS | low P | Yield | fallow | pre-crop | 5 | 5 | -1.72 | 0.085 | 0.085 | ns |
| Grinkan | WW | high P | Yield | fallow | pre-crop | 5 | 5 | 2.61 | 0.009 | 0.009 | ** |
| Grinkan | WW | low P | Yield | fallow | pre-crop | 4 | 3 | 1.06 | 0.289 | 0.289 | ns |
| Grinkan | WS | high P | Yield | fallow | pre-crop | 4 | 5 | -0.735 | 0.462 | 0.462 | ns |
| Grinkan | WS | low P | Yield | fallow | pre-crop | 3 | 5 | -0.447 | 0.655 | 0.655 | ns |
| M-35 | WW | high P | Yield | fallow | pre-crop | 5 | 5 | 0.94 | 0.347 | 0.347 | ns |
| M-35 | WW | low P | Yield | fallow | pre-crop | 5 | 4 | 0.49 | 0.624 | 0.624 | ns |
| M-35 | WS | high P | Yield | fallow | pre-crop | 5 | 5 | -1.53 | 0.126 | 0.126 | ns |
| M-35 | WS | low P | Yield | fallow | pre-crop | 4 | 5 | 1.34 | 0.18 | 0.18 | ns |

Table S5 Dunn’s Multiple Comparison between five sorghum genotypes

| **Water l.** | **P l.** | **Crotation** | **.y.** | **group1** | **group2** | **n1** | **n2** | **statistic** | **p** | **p.adj** | **p.adj.signif** |
| --- | --- | --- | --- | --- | --- | --- | --- | --- | --- | --- | --- |
| WS | high P | fallow | Yield | CSV14R | Keslapoor | 5 | 5 | -1.25 | 0.21 | 0.21 | ns |
| WS | high P | fallow | Yield | CSV14R | Lata 3 | 5 | 5 | 1.75 | 0.0809 | 0.0809 | ns |
| WS | high P | fallow | Yield | CSV14R | Grinkan | 5 | 4 | 1.24 | 0.213 | 0.213 | ns |
| WS | high P | fallow | Yield | CSV14R | M-35 | 5 | 5 | -0.582 | 0.561 | 0.561 | ns |
| WS | high P | fallow | Yield | Keslapoor | Lata 3 | 5 | 5 | 3 | 0.0027 | 0.00271 | ** |
| WS | high P | fallow | Yield | Keslapoor | Grinkan | 5 | 4 | 2.43 | 0.0152 | 0.0152 | * |
| WS | high P | fallow | Yield | Keslapoor | M-35 | 5 | 5 | 0.671 | 0.502 | 0.502 | ns |
| WS | high P | fallow | Yield | Lata 3 | Grinkan | 5 | 4 | -0.401 | 0.688 | 0.688 | ns |
| WS | high P | fallow | Yield | Lata 3 | M-35 | 5 | 5 | -2.33 | 0.0199 | 0.0199 | * |
| WS | high P | fallow | Yield | Grinkan | M-35 | 4 | 5 | -1.79 | 0.0729 | 0.0729 | ns |
| WS | high P | pre-crop | Yield | CSV14R | Keslapoor | 4 | 5 | 0.0169 | 0.987 | 0.987 | ns |
| WS | high P | pre-crop | Yield | CSV14R | Lata 3 | 4 | 5 | 2.02 | 0.0432 | 0.0432 | * |
| WS | high P | pre-crop | Yield | CSV14R | Grinkan | 4 | 5 | 2.72 | 0.0065 | 0.00652 | ** |
| WS | high P | pre-crop | Yield | CSV14R | M-35 | 4 | 5 | -0.0282 | 0.978 | 0.978 | ns |
| WS | high P | pre-crop | Yield | Keslapoor | Lata 3 | 5 | 5 | 2.13 | 0.0334 | 0.0334 | * |
| WS | high P | pre-crop | Yield | Keslapoor | Grinkan | 5 | 5 | 2.87 | 0.0041 | 0.00414 | ** |
| WS | high P | pre-crop | Yield | Keslapoor | M-35 | 5 | 5 | -0.0478 | 0.962 | 0.962 | ns |
| WS | high P | pre-crop | Yield | Lata 3 | Grinkan | 5 | 5 | 0.741 | 0.459 | 0.459 | ns |
| WS | high P | pre-crop | Yield | Lata 3 | M-35 | 5 | 5 | -2.17 | 0.0297 | 0.0297 | * |
| WS | high P | pre-crop | Yield | Grinkan | M-35 | 5 | 5 | -2.92 | 0.0036 | 0.00355 | ** |
| WS | low P | fallow | Yield | CSV14R | Keslapoor | 6 | 5 | -1.86 | 0.063 | 0.063 | ns |
| WS | low P | fallow | Yield | CSV14R | Lata 3 | 6 | 5 | 0.301 | 0.764 | 0.764 | ns |
| WS | low P | fallow | Yield | CSV14R | Grinkan | 6 | 3 | 1.47 | 0.142 | 0.142 | ns |
| WS | low P | fallow | Yield | CSV14R | M-35 | 6 | 4 | -2.01 | 0.0449 | 0.0449 | * |
| WS | low P | fallow | Yield | Keslapoor | Lata 3 | 5 | 5 | 2.07 | 0.0387 | 0.0387 | * |
| WS | low P | fallow | Yield | Keslapoor | Grinkan | 5 | 3 | 2.96 | 0.003 | 0.00304 | ** |
| WS | low P | fallow | Yield | Keslapoor | M-35 | 5 | 4 | -0.252 | 0.801 | 0.801 | ns |
| WS | low P | fallow | Yield | Lata 3 | Grinkan | 5 | 3 | 1.17 | 0.241 | 0.241 | ns |
| WS | low P | fallow | Yield | Lata 3 | M-35 | 5 | 4 | -2.2 | 0.0277 | 0.0277 | * |
| WS | low P | fallow | Yield | Grinkan | M-35 | 3 | 4 | -3.06 | 0.0023 | 0.00225 | ** |
| WS | low P | pre-crop | Yield | CSV14R | Keslapoor | 5 | 5 | -0.0473 | 0.962 | 0.962 | ns |
| WS | low P | pre-crop | Yield | CSV14R | Lata 3 | 5 | 5 | 0.733 | 0.463 | 0.463 | ns |
| WS | low P | pre-crop | Yield | CSV14R | Grinkan | 5 | 5 | 3.12 | 0.0018 | 0.0018 | ** |
| WS | low P | pre-crop | Yield | CSV14R | M-35 | 5 | 5 | 0.449 | 0.653 | 0.653 | ns |
| WS | low P | pre-crop | Yield | Keslapoor | Lata 3 | 5 | 5 | 0.781 | 0.435 | 0.435 | ns |
| WS | low P | pre-crop | Yield | Keslapoor | Grinkan | 5 | 5 | 3.17 | 0.0015 | 0.00153 | ** |
| WS | low P | pre-crop | Yield | Keslapoor | M-35 | 5 | 5 | 0.497 | 0.619 | 0.619 | ns |
| WS | low P | pre-crop | Yield | Lata 3 | Grinkan | 5 | 5 | 2.39 | 0.0169 | 0.0169 | * |
| WS | low P | pre-crop | Yield | Lata 3 | M-35 | 5 | 5 | -0.284 | 0.777 | 0.777 | ns |
| WS | low P | pre-crop | Yield | Grinkan | M-35 | 5 | 5 | -2.67 | 0.0075 | 0.00752 | ** |
| WW | high P | fallow | Yield | CSV14R | Keslapoor | 4 | 6 | 0.0548 | 0.956 | 0.956 | ns |
| WW | high P | fallow | Yield | CSV14R | Lata 3 | 4 | 4 | -1.95 | 0.0512 | 0.0512 | ns |
| WW | high P | fallow | Yield | CSV14R | Grinkan | 4 | 5 | -2.14 | 0.0324 | 0.0324 | * |
| WW | high P | fallow | Yield | CSV14R | M-35 | 4 | 5 | -2.6 | 0.0092 | 0.00922 | ** |
| WW | high P | fallow | Yield | Keslapoor | Lata 3 | 6 | 4 | -2.19 | 0.0285 | 0.0285 | * |
| WW | high P | fallow | Yield | Keslapoor | Grinkan | 6 | 5 | -2.43 | 0.0151 | 0.0151 | * |
| WW | high P | fallow | Yield | Keslapoor | M-35 | 6 | 5 | -2.94 | 0.0033 | 0.00325 | ** |
| WW | high P | fallow | Yield | Lata 3 | Grinkan | 4 | 5 | -0.0843 | 0.933 | 0.933 | ns |
| WW | high P | fallow | Yield | Lata 3 | M-35 | 4 | 5 | -0.548 | 0.584 | 0.584 | ns |
| WW | high P | fallow | Yield | Grinkan | M-35 | 5 | 5 | -0.492 | 0.623 | 0.623 | ns |
| WW | high P | pre-crop | Yield | CSV14R | Keslapoor | 5 | 4 | 0.162 | 0.871 | 0.871 | ns |
| WW | high P | pre-crop | Yield | CSV14R | Lata 3 | 5 | 6 | -2.25 | 0.0244 | 0.0244 | * |
| WW | high P | pre-crop | Yield | CSV14R | Grinkan | 5 | 5 | -0.0859 | 0.932 | 0.932 | ns |
| WW | high P | pre-crop | Yield | CSV14R | M-35 | 5 | 5 | -0.902 | 0.367 | 0.367 | ns |
| WW | high P | pre-crop | Yield | Keslapoor | Lata 3 | 4 | 6 | -2.28 | 0.0226 | 0.0226 | * |
| WW | high P | pre-crop | Yield | Keslapoor | Grinkan | 4 | 5 | -0.243 | 0.808 | 0.808 | ns |
| WW | high P | pre-crop | Yield | Keslapoor | M-35 | 4 | 5 | -1.01 | 0.311 | 0.311 | ns |
| WW | high P | pre-crop | Yield | Lata 3 | Grinkan | 6 | 5 | 2.16 | 0.0306 | 0.0306 | * |
| WW | high P | pre-crop | Yield | Lata 3 | M-35 | 6 | 5 | 1.31 | 0.191 | 0.191 | ns |
| WW | high P | pre-crop | Yield | Grinkan | M-35 | 5 | 5 | -0.816 | 0.414 | 0.414 | ns |
| WW | low P | fallow | Yield | CSV14R | Keslapoor | 4 | 5 | -0.022 | 0.982 | 0.982 | ns |
| WW | low P | fallow | Yield | CSV14R | Lata 3 | 4 | 5 | -2.26 | 0.0236 | 0.0236 | * |
| WW | low P | fallow | Yield | CSV14R | Grinkan | 4 | 4 | -1.51 | 0.131 | 0.131 | ns |
| WW | low P | fallow | Yield | CSV14R | M-35 | 4 | 5 | -0.989 | 0.323 | 0.323 | ns |
| WW | low P | fallow | Yield | Keslapoor | Lata 3 | 5 | 5 | -2.38 | 0.0174 | 0.0174 | * |
| WW | low P | fallow | Yield | Keslapoor | Grinkan | 5 | 4 | -1.57 | 0.116 | 0.116 | ns |
| WW | low P | fallow | Yield | Keslapoor | M-35 | 5 | 5 | -1.03 | 0.305 | 0.305 | ns |
| WW | low P | fallow | Yield | Lata 3 | Grinkan | 5 | 4 | 0.67 | 0.503 | 0.503 | ns |
| WW | low P | fallow | Yield | Lata 3 | M-35 | 5 | 5 | 1.35 | 0.176 | 0.176 | ns |
| WW | low P | fallow | Yield | Grinkan | M-35 | 4 | 5 | 0.604 | 0.546 | 0.546 | ns |
| WW | low P | pre-crop | Yield | CSV14R | Keslapoor | 5 | 5 | -0.146 | 0.884 | 0.884 | ns |
| WW | low P | pre-crop | Yield | CSV14R | Lata 3 | 5 | 5 | -1.22 | 0.223 | 0.223 | ns |
| WW | low P | pre-crop | Yield | CSV14R | Grinkan | 5 | 3 | -0.169 | 0.866 | 0.866 | ns |
| WW | low P | pre-crop | Yield | CSV14R | M-35 | 5 | 4 | -1.16 | 0.246 | 0.246 | ns |
| WW | low P | pre-crop | Yield | Keslapoor | Lata 3 | 5 | 5 | -1.07 | 0.284 | 0.284 | ns |
| WW | low P | pre-crop | Yield | Keslapoor | Grinkan | 5 | 3 | -0.0422 | 0.966 | 0.966 | ns |
| WW | low P | pre-crop | Yield | Keslapoor | M-35 | 5 | 4 | -1.02 | 0.307 | 0.307 | ns |
| WW | low P | pre-crop | Yield | Lata 3 | Grinkan | 5 | 3 | 0.886 | 0.376 | 0.376 | ns |
| WW | low P | pre-crop | Yield | Lata 3 | M-35 | 5 | 4 | -0.0115 | 0.991 | 0.991 | ns |
| WW | low P | pre-crop | Yield | Grinkan | M-35 | 3 | 4 | -0.857 | 0.391 | 0.391 | ns |
|  |  |  |  |  |  |  |  |  |  |  |  |

Table S6: Yield: Dunn’s Multiple Comparison between P levels

| **Nsource** | **Water l.** | **.y.** | **group1** | **group2** | **n1** | **n2** | **statistic** | **p** | **p.adj** | **p.adj.signif** |
| --- | --- | --- | --- | --- | --- | --- | --- | --- | --- | --- |
| fallow | WW | Yield | high P | low P | 24 | 23 | 0 | 1 | 1 | ns |
| fallow | WS | Yield | high P | low P | 24 | 23 | -2.17 | 0.03 | 0.03 | * |
| pre-crop | WW | Yield | high P | low P | 25 | 22 | -0.917 | 0.359 | 0.359 | ns |
| pre-crop | WS | Yield | high P | low P | 24 | 25 | -0.781 | 0.435 | 0.435 | ns |

Table S7 Root-to-Shoot ratio: Dunn’s Multiple Comparison between P levels

| **Development Stage** | **Genotype** | **.y.** | **high P mean** | **high P se** | **low P mean** | **low P se** | **high P n1** | **low P n2** | **stat.** | **p** | **p.adj** | **p.adj.signif** |
| --- | --- | --- | --- | --- | --- | --- | --- | --- | --- | --- | --- | --- |
| Stem E. | Early | RSRatio | 0.14 | ±0.050 | 0.255 | ±0.014 | 4 | 4 | 2.12 | 0.034 | 0.034 | * |
| Stem E. | Late | RSRatio | 0.156 | ±0.028 | 0.147 | ±0.037 | 6 | 6 | 0.16 | 0.873 | 0.873 | ns |
| Anthesis | Early | RSRatio | 0.433 | ±0.010 | 0.455 | ±0.058 | 4 | 4 | 0.00 | 1.000 | 1.000 | ns |
| Anthesis | Late | RSRatio | 0.286 | ±0.048 | 0.245 | ±0.021 | 6 | 6 | 1.44 | 0.150 | 0.150 | ns |
| Maturity | Early | RSRatio | 0.233 | ±0.022 | 0.319 | ±0.013 | 4 | 4 | 2.31 | 0.021 | 0.021 | * |
| Maturity | Late | RSRatio | 0.244 | ±0.020 | 0.347 | ±0.056 | 6 | 6 | 1.44 | 0.150 | 0.150 | ns |

Figure S10 Experimental set up
